# Atomic layer confined vacancies for atomic-level insights into carbon dioxide electroreduction

**DOI:** 10.1038/ncomms14503

**Published:** 2017-02-21

**Authors:** Shan Gao, Zhongti Sun, Wei Liu, Xingchen Jiao, Xiaolong Zu, Qitao Hu, Yongfu Sun, Tao Yao, Wenhua Zhang, Shiqiang Wei, Yi Xie

**Affiliations:** 1 Hefei National Laboratory for Physical Sciences at Microscale, CAS Center for Excellence in Nanoscience, University of Science and Technology of China, Hefei 230026, China; 2 National Synchrotron Radiation Laboratory, University of Science and Technology of China, Hefei, Anhui 230029, China; 3 Hefei Science Center of CAS, Hefei, Anhui 230061, China

## Abstract

The role of oxygen vacancies in carbon dioxide electroreduction remains somewhat unclear. Here we construct a model of oxygen vacancies confined in atomic layer, taking the synthetic oxygen-deficient cobalt oxide single-unit-cell layers as an example. Density functional theory calculations demonstrate the main defect is the oxygen(II) vacancy, while X-ray absorption fine structure spectroscopy reveals their distinct oxygen vacancy concentrations. Proton transfer is theoretically/experimentally demonstrated to be a rate-limiting step, while energy calculations unveil that the presence of oxygen(II) vacancies lower the rate-limiting activation barrier from 0.51 to 0.40 eV via stabilizing the formate anion radical intermediate, confirmed by the lowered onset potential from 0.81 to 0.78 V and decreased Tafel slope from 48 to 37 mV dec^−1^. Hence, vacancy-rich cobalt oxide single-unit-cell layers exhibit current densities of 2.7 mA cm^−2^ with ca. 85% formate selectivity during 40-h tests. This work establishes a clear atomic-level correlation between oxygen vacancies and carbon dioxide electroreduction.

## Abstract (web summary)

The role of oxygen vacancies in carbon dioxide reduction remains somewhat unclear. Here, the authors fabricate vacancy-rich and vacancy-poor Co_3_O_4_ single-unit-cell layers, and demonstrate by X-ray absorption and DFT that the material is a promising platform for mechanistic studies of carbon dioxide reduction.

Motivated by the increasing trepidations about CO_2_-induced global warming and depletion of the finite fossil fuel resources, developing renewable energy alternatives epitomizes one of the major scientific challenges for the twenty-first century^1^,^2^,^3^,^4^. In this current scenario, electrochemical CO_2_ reduction into hydrocarbon fuels is considered as a potentially ‘clean' approach for attaining fuels and bulk chemicals that are usually derived from oil or natural gas^4^,^5^,^6^,^7^,^8^,^9^. Electrocatalytic CO_2_ reduction mainly encompasses of the following elementary steps: (1) CO_2_ adsorption on active sites; (2) activation of CO_2_ to form 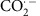 or 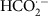 or other intermediates; (3) dissociation of C–O bond comprising the participation of protons and electron transfer (one, two or multiple electron process); (4) desorption of reduced products from the active sites^1^,^3^,^4^,^9^,^10^,^11^. In relation to these, the most critical bottleneck in developing efficient CO_2_ electroreduction lies in the chemical activation of CO_2_ (refs 4, 9), which usually entails high overpotentials and instigates the formation of excess competitive reduction products such as H_2_, thus bringing about in low energetic efficiency and poor product selectivity^12^,^13^. Therefore, lowering the activation energy barrier of CO_2_ holds the key to a major breakthrough in electrocatalytic CO_2_ reduction.

Recently, oxygen vacancies in oxides have been reported to promote CO_2_ activation and dissociation processes by means of tailoring their electronic structures, charge transport and surface properties^14^,^15^. The presence of oxygen vacancies decorates the surface as electron-rich, while the excess electrons indulge CO_2_ adsorption and activation^16^,^17^,^18^,^19^. For instance, Zapol *et al*. demonstrate that the reduced (101) surface of anatase TiO_2_ is considerably more auspicious for CO_2_ adsorption accompanying charge transfer to CO_2_ molecules for forming 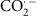 species in comparison with the oxidized surface^18^. In addition, Li *et al*. report that the formed 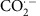 intermediate could be spontaneously dissociated into CO even in the dark on a partially oxygen-depleted Cu(I)/TiO_2−*x*_ surface^19^. However, to date, atomic-level comprehensions on the role of oxygen vacancies during CO_2_ reduction is still at infant stage. This is primarily credited to the following two reasons: (1) oxygen vacancies are usually present on the interior of catalysts rather than on the surface, and hence they may possibly not effectively embroil the catalytic reactions^20^; and (2) the presence of abundant microstructures such as interface, and capping agents, could adversely affect or cover the effect of oxygen vacancies on CO_2_ reduction activity^21^. To gain in-depth atomic-level understanding on the correlation between oxygen vacancies and CO_2_ reduction property, it would be rather vital to simplify the catalyst model and conduit it with the real catalyst containing oxygen vacancies.

Herein, we initially construct an ideal and simple model of intact oxide-based atomic layer and hence deliberately create oxygen vacancies on the surface, with efforts to disclose atomic-level insights between oxygen vacancies and CO_2_ reduction catalysis. The atomic thickness not only favours building clear atomic structure^22^, but also enables the majority of oxygen vacancies distribution on the surface. In this regard, a model of Co_3_O_4_ atomic layer with oxygen vacancies would be a promising candidate, thanks to its wide applications in catalysis as well as its environmental friendliness, abundance of reserves and favourable thermal stability^9^,^23^,^24^. However, for non-layered compounds, especially for cubic Co_3_O_4_ without anisotropy, fabrication of its atomic layer is particularly a daring task owing to the hard breakage of strong in-plane bonds and the lack of intrinsic driving force for two-dimensional anisotropic growth, let alone the designed synthesis of Co_3_O_4_ atomic layer with well-controlled oxygen vacancies.

## Results

### Characterizations for Co_3_O_4_ single-unit-cell layers

**Figure 1:**
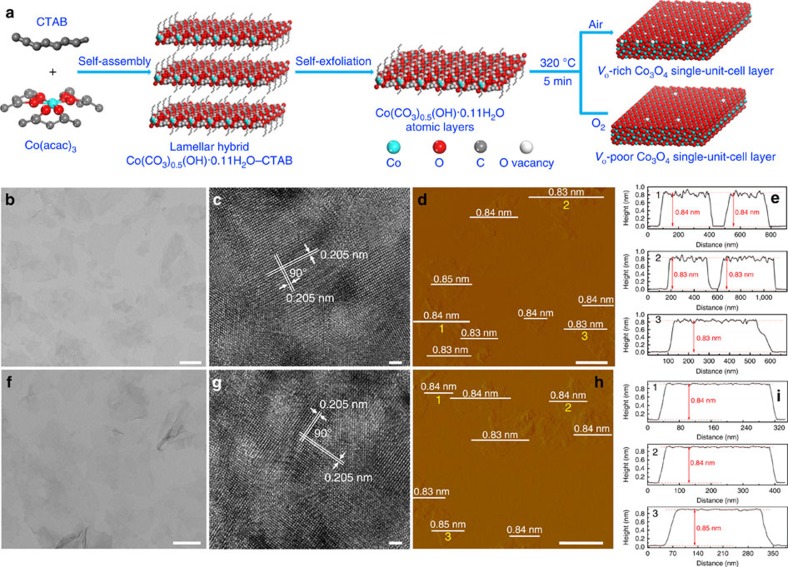
Preparation and characterization for the *V*_o_-rich and *V*_o_-poor Co_3_O_4_ single-unit-cell layer. (**a**) Scheme for the formation of *V*_o_-rich and *V*_o_-poor Co_3_O_4_ single-unit-cell layer, respectively. Characterization for the *V*_o_-rich Co_3_O_4_ single-unit-cell layer: (**b**) TEM image, (**c**) HRTEM image, (**d**) AFM image and (**e**) the corresponding height profiles; the numbers from 1 to 3 in **d** correspond to the numbers from 1 to 3 in **e**. Characterization for the *V*_o_-poor Co_3_O_4_ single-unit-cell layer: (**f**) TEM image, (**g**) HRTEM image. (**h**) AFM image and (**i**) the corresponding height profiles; the numbers from 1 to 3 in **i** corresponding to the numbers from 1 to 3 in **h**. The scale bars in **b**–**d** and **f**–**h** are 250, 1, 500, 200, 1 and 500 nm, respectively.

**Figure 2:**
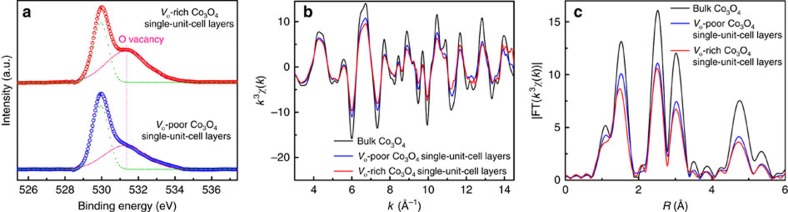
XPS spectra and synchrotron radiation XAFS measurements. (**a**) O 1*s* XPS spectra of *V*_o_-rich and *V*_o_-poor Co_3_O_4_ single-unit-cell layers. (**b**) Co K-edge extended XAFS oscillation function *k*^3^*χ*(*k*). (**c**) The corresponding Fourier transforms FT(*k*^3^*χ*(*k*)).

To achieve the above important goal, *V*_o_-rich and *V*_o_-poor Co_3_O_4_ single-unit-cell layers were successfully fabricated via a lamellar inorganic–organic hybrid intermediate strategy (Fig. 1a). Initially, a lamellar Co(CO_3_)_0.5_(OH)·0.11H_2_O–CTAB hybrid was synthesized via a self-assembly process between Co(acac)_3_ and CTAB, in which the ordered mesostructure was verified by the corresponding small-angle X-ray diffraction pattern obtained at 180 °C for 12 h (Supplementary Fig. 1a). With the reaction time extending to 20 h, the lamellar Co(CO_3_)_0.5_(OH)·0.11H_2_O–CTAB hybrid gradually self-exfoliates into the ultrathin Co(CO_3_)_0.5_(OH)·0.11H_2_O layers, confirmed by the corresponding X-ray diffraction, transmission electron microscopy (TEM) and atomic force microscopy (AFM) characterizations in Supplementary Fig. 1. Then, the following fast-heating process in distinct air and O_2_ atmospheres resulted in the successful formation of Co_3_O_4_ single-unit-cell layers with different concentrations of oxygen vacancies. Taking the products obtained at 320 °C for 5 min in air as an example, their X-ray diffraction pattern for the accumulated powder sample could be readily indexed to cubic Co_3_O_4_ (JCPDS No. 78–1969), further verified by the corresponding Raman spectra (Supplementary Fig. 2a,b)^25^. In addition, their X-ray photoelectron spectra (XPS) spectra in Supplementary Fig. 2d,e demonstrated the formation of pure Co_3_O_4_, further confirmed by the corresponding infrared spectrum in Supplementary Fig. 2c, indicating the absence of impurities such as CTAB on the surface of the as-obtained sample^26^. TEM image in Fig. 1b reveals their sheet-like morphology, while the nearly transparent feature indicates their ultrathin thickness. The high-resolution TEM image in Fig. 1c illustrates their [001] orientation, while the AFM image and the corresponding height profiles in Fig. 1d,e reveal their average 0.84 nm thickness, which fairly agrees with the thickness of one unit cell along the [001] direction. More importantly, their O 1*s* core level spectrum in Fig. 2a clearly showed two distinct peaks: one peak at 529.8 eV was deemed as the lattice oxygen, while the other one located at 531.4 eV could be ascribed to the oxygen atoms in the vicinity of an oxygen vacancy^24^,^27^. However, their peak area of 531.4 eV is widely different with that calcinated at 320 °C for 5 min in the O_2_ atmosphere (Fig. 1f–i), which indicates that the ultrathin Co_3_O_4_ sheets obtained in the air atmosphere possess larger concentration of oxygen vacancies than those obtained in the O_2_ atmosphere. Therefore, all the above results proved the succssful synthesis of Co_3_O_4_ single-unit-cell layers with distinct oxygen vacancy concentrations, thus providing the ideal material models to study the relationship between oxygen vacancies and CO_2_ reduction activity.

### Synchrotron radiation XAFS measurements

To further disclose the distinct oxygen vacancy concentrations in those fabricated Co_3_O_4_ samples, X-ray absorption fine structure spectroscopy (XAFS) measurements at Co *K*-edge were carried out at 1W1B station in BSRF (Beijing Synchrotron Radiation Facility, China). As shown by the raw Co *K*-edge EXAFS data in Supplementary Fig. 3, the post-edge oscillation amplitude for the *V*_o_-rich Co_3_O_4_ single-unit-cell layers exhibited obvious differences in comparison with the *V*_o_-poor Co_3_O_4_ single-unit-cell layers and bulk counterpart, further confirmed by their corresponding Co *K*-edge *k*^3^*χ*(*k*) oscillation curve and Fourier transformed *k*^3^*χ*(*k*) functions (Fig. 2b,c), qualitatively revealing their distinct local atomic arrangement. To obtain quantitative structural parameters around Co atoms confined in the Co_3_O_4_ single-unit-cell layers, a least-squares curve fitting was conducted and the EXAFS data fitting results were shown in Table 1 and Supplementary Fig. 4. For the *V*_o_-poor Co_3_O_4_ single-unit-cell layers, the coordination numbers for Co-O, Co-Co_1_, Co-O_1_, Co-Co_2_ and Co-O_2_ coordinations reduced, while their disorder degrees increased compared with bulk counterpart, which implied the presence of many dangling bonds as well as an obvious distortion on their surface. The surface distortion in turn helped to endow them with excellent structural stability^24^,^28^,^29^. More importantly, the coordination numbers for Co-O, Co-O_1_ and Co-O_2_ coordinations, confined in the *V*_o_-rich Co_3_O_4_ single-unit-cell layers, further decreased as compared with the *V*_o_-poor Co_3_O_4_ single-unit-cell layers, while their coordination numbers for Co-Co_1_ and Co-Co_2_ coordinations did not show any noticeable variation (Table 1), which indicated the former's higher concentration of oxygen vacancies. Thus, the EXAFS results clearly demonstrated the distinct oxygen vacancy concentrations in the synthesized two samples of Co_3_O_4_ single-unit-cell layers, fairly agreeing with that of the O 1*s* XPS spectra in Fig. 2a, and the results revealed by inductively coupled plasma atomic emission spectroscopy and titration method (see details in Methods section).

### Electrocatalytic reduction of CO_2_ into formate

**Figure 3:**
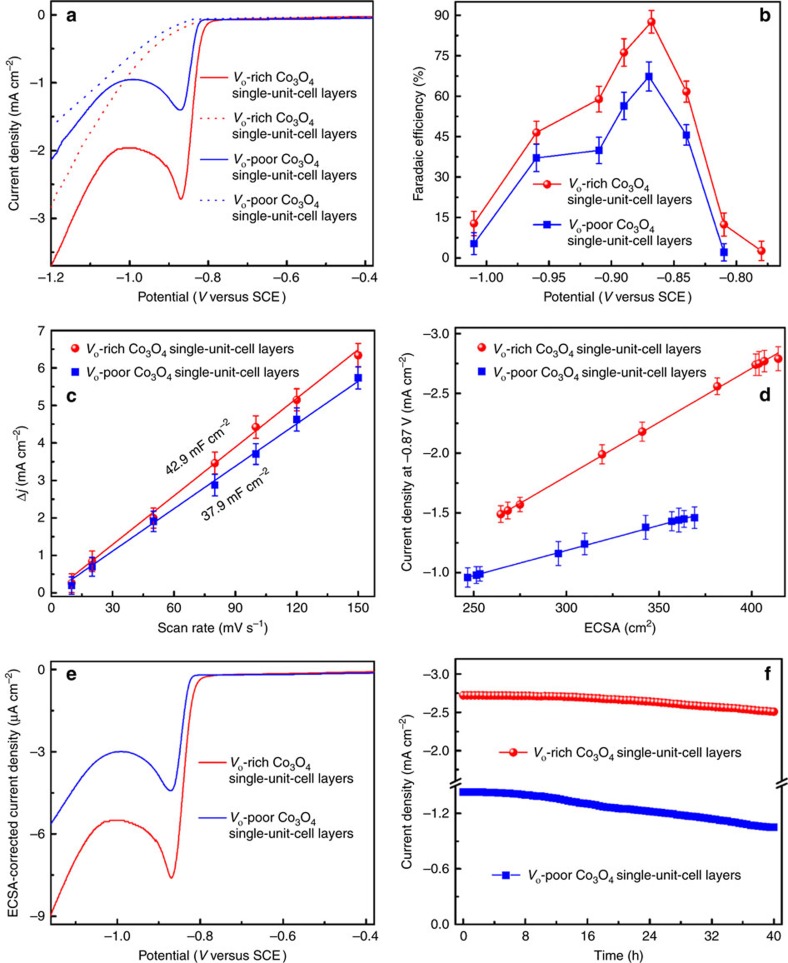
Electrocatalytic reduction of CO_2_ into formate by the *V*_o_-rich and *V*_o_-poor Co_3_O_4_ single-unit-cell layers. (**a**) Linear sweep voltammetric curves in a CO_2_-saturated (solid line) and N_2_-saturated (dashed line) 0.1 M KHCO_3_ aqueous solution; (**b**) Faradaic efficiencies of formate at different applied potentials; (**c**) charging current density differences plotted against scan rates; (**d**) current density at −0.87 V versus SCE plotted against ECSA for various material at different loadings; (**e**) ECSA-corrected current densities versus applied potentials; (**f**) Chronoamperometry results at the potential of −0.87 V versus SCE. The error bars in **b**–**d** represent the s.d.'s of five independent measurements of the same sample.

To give evidences of the correlation between oxygen vacancies and CO_2_ reduction activity, the potentiodynamic electrochemical behaviours for the *V*_o_-rich and *V*_o_-poor Co_3_O_4_ single-unit-cell layers were investigated in CO_2_-saturated 0.1 M KHCO_3_ solution. As shown by the linear sweep voltammetry (LSV) in Fig. 3a, the large cathodic peaks appeared at ca. −0.87 V versus saturated calomel electrode (SCE) could be attributed to the catalytic CO_2_ reduction, since no reduction peaks were observed in the corresponding N_2_-saturated 0.1 M KHCO_3_ solution. For instance, the *V*_o_-rich Co_3_O_4_ single-unit-cell layers exhibited a current density of 2.7 mA cm^−2^ at −0.87 V versus SCE, roughly two times as large as that of the *V*_o_-poor Co_3_O_4_ single-unit-cell layers, strongly demonstrating the significant role of oxygen vacancies in improving CO_2_ electroreduction activity. It is noticeable that the cathodic reduction peak at ca. −0.87 V versus SCE may be closely related to the reduction of CO_2_ into formate in the electrolyte^4^. To further pinpoint the reduction products, stepped-potential electrolyses at each given potential for 4 h were performed to quantify the liquid and gas products by ^1^H nuclear magnetic resonance and gas chromatography analysis. The results in Fig. 3b revealed that the *V*_o_-rich Co_3_O_4_ single-unit-cell layers possessed a maximum faradaic efficiency of 87.6% for producing formate at a moderately negative potential of −0.87 V versus SCE, while the *V*_o_-poor Co_3_O_4_ single-unit-cell layers showed a faradaic efficiency of 67.3%, further demonstrating the former's superior selectivity for formate production. Moreover, as shown in Supplementary Fig. 5, both the *V*_o_-rich and *V*_o_-poor Co_3_O_4_ single-unit-cell layers produced the gas products of H_2_, CO and CH_4_ with different selectivities at different potentials, in which the faradaic efficiencies of CO and CH_4_ for the *V*_o_-rich Co_3_O_4_ single-unit-cell layers were still higher than those of the *V*_o_-poor Co_3_O_4_ single-unit-cell layers, further confirming the superior activity induced by abundant oxygen vacancies. Importantly, one can also see that at moderate applied potentials, the main CO_2_ reduction product was the formate for both the *V*_o_-rich and *V*_o_-poor Co_3_O_4_ single-unit-cell layers. In addition, Fig. 3b also illustrates that the *V*_o_-rich Co_3_O_4_ single-unit-cell layers attained an onset potential of −0.78 V versus SCE, which was smaller than −0.81 V versus SCE for the *V*_o_-poor Co_3_O_4_ single-unit-cell layers, confirming the high activity of *V*_o_-rich Co_3_O_4_ single-unit-cell layers^30^. Furthermore, the LSV curves in N_2_-saturated 0.1 M KHCO_3_ solution further indicated that the *V*_o_-rich Co_3_O_4_ single-unit-cell layers also possessed increased H_2_O reduction activity relative to the *V*_o_-poor Co_3_O_4_ single-unit-cell layers, especially at very negative potentials, which in turn implied the higher catalytic activity induced by the abundant oxygen vacancies. Thus, the above results demonstrated that the *V*_o_-rich Co_3_O_4_ single-unit-cell layers possessed relatively higher activity and selectivity towards CO_2_ electroreduction into formate compared with the *V*_o_-poor Co_3_O_4_ single-unit-cell layers.

To disclose the crucial factors in affecting the catalytic performances, electrochemical surface area (ECSA) of these catalysts was determined by measuring the double-layer capacitance (Fig. 3c). Interestingly, the *V*_o_-rich Co_3_O_4_ single-unit-cell layers exhibited nearly the same ECSA as that of the *V*_o_-poor Co_3_O_4_ single-unit-cell layers, which cannot account for the former's two times higher catalytic activity. This indicates that the superior catalytic performance of the *V*_o_-rich Co_3_O_4_ single-unit-cell layers is not due to the ECSA, but instead the increased concentration of oxygen vacancies, which favour much higher activity and selectivity towards formate production. In addition, the better intrinsic catalytic activity for the *V*_o_-rich Co_3_O_4_ single-unit-cell layers could be further verified by their higher slope of catalytic activity versus ECSA, and also by their ECSA-corrected current densities in comparison with the *V*_o_-poor Co_3_O_4_ single-unit-cell layers (Fig. 3d,e). Moreover, stability is another significant criterion to evaluate a catalyst and hence to test the stability of catalyst continuous CO_2_ reduction at −0.87 V versus SCE was conducted for probing the durability of the above electrocatalysts. The *V*_o_-rich Co_3_O_4_ single-unit-cell layers showed negligible decay in the steady-state current density and their Faradaic efficiency for producing formate was always >85% during the tested period of 40 h (Fig. 3f; Supplementary Fig. 6), suggesting their very favourable stability, further confirmed by their corresponding post-reaction analysis in Supplementary Figs 7 and 8. In contrast, the *V*_o_-poor Co_3_O_4_ single-unit-cell layers possessed relatively poor long-term stability with Faradaic efficiency down to ca. 65%.

## Discussion

Notably, the promoted CO_2_ reduction activity and selectivity could be primarily ascribed to the oxygen vacancies confined in Co_3_O_4_ single-unit-cell layers, in which the confined oxygen vacancies could serve as the active sites for stabilizing the reduction intermediates and hence lowering the activation energy barrier. Here it is suggested that CO_2_ molecules are initially adsorbed on the surface of catalysts and hence undergo the following reaction steps during its reduction into formate:


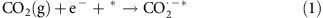



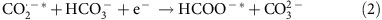



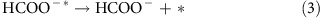


where the asterisk denotes a catalytically active site and the whole reaction can be written as


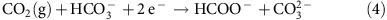


**Figure 4:**
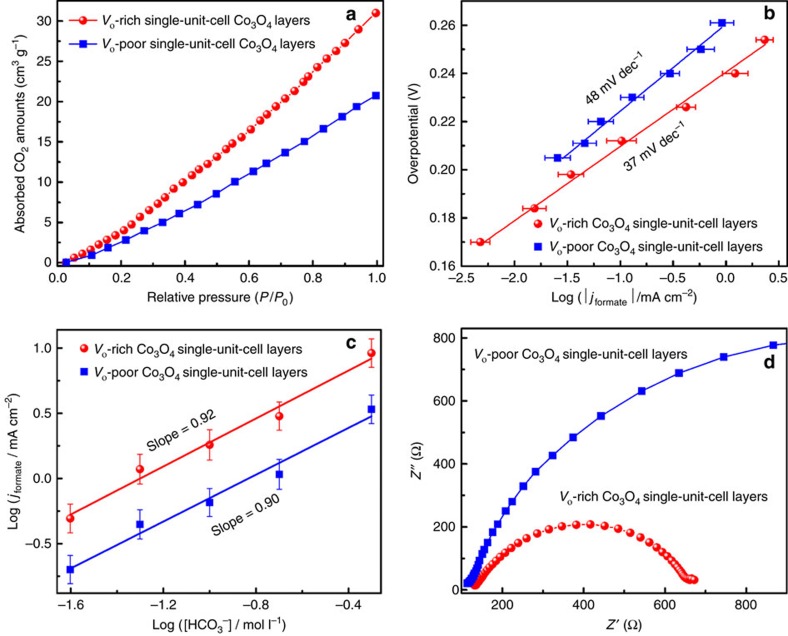
Comparison of CO_2_ adsorption amount, Tafel plots and electrokinetics. (**a**) CO_2_ adsorption isotherms, (**b**) Tafel plots, (**c**) partial current density of formate production versus 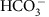 concentration at a constant potential of −0.87 V versus SCE and (**d**) electrochemical impedance spectra for the *V*_o_-rich and *V*_o_-poor Co_3_O_4_ single-unit-cell layers. The error bars in **b** and **c** represent the s.d.'s of five independent measurements of the same sample.

In other words, the adsorption process of CO_2_ molecules plays a vital role in affecting the reduction activity. In this case, the volumetric CO_2_ adsorption measurement was carried out, and the results in Fig. 4a revealed that the *V*_o_-rich Co_3_O_4_ single-unit-cell layers exhibited a higher CO_2_ adsorption capacity than the *V*_o_-poor Co_3_O_4_ single-unit-cell layers, indicating that the higher oxygen vacancy concentration allowed for increased CO_2_ adsorption. Moreover, the additional electrolysis data provided some insights into the mechanisms underlying CO_2_ reduction into formate for the above two samples. As shown in Fig. 4b, the *V*_o_-rich and *V*_o_-poor Co_3_O_4_ single-unit-cell layers possessed the Tafel slopes of 37 and 48 mV dec^−1^, respectively. Note that the Tafel slopes close to 59 mV dec^−1^ supported a possible reduction mechanism that involved a chemical rate-determining H^+^ transfer step^1^,^13^,^31^,^32^. To disclose whether the H^+^ transfer was the rate-limiting step, we further performed the electrolyses at a constant applied potential with 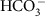 concentrations ranging from 0.5 to 0.025 M, with KClO_4_ added to the electrolyte to maintain ionic strength. As shown by the log(*j*_formate_) versus log([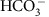]) plots in Fig. 4c, the *V*_o_-rich and *V*_o_-poor Co_3_O_4_ single-unit-cell layers exhibited the slopes of 0.92 and 0.90, respectively, indicating approximate first-order dependence of the reaction rate on the concentration of 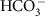. To verify whether the proton donation process from 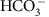 was the rate-limiting step, we performed the corresponding theoretical analysis and the details were shown in Supplementary Methods. The results clearly demonstrated that the reaction rate for formate product showed the first-order dependence on the concentration of 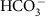, only based on the assumption that the second step (2) was the rate-limiting step. The theoretical analysis as well as the experimental results synergistically confirmed that H^+^ donation from 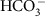 was indeed the rate-limiting step for both the *V*_o_-rich and *V*_o_-poor Co_3_O_4_ single-unit-cell layers. In addition, to disclose the surface coverage of possible reaction intermediates, we further conducted the theoretical analysis based on the experimental Tafel slopes and the log(*j*_formate_) versus log([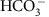]) plots in Fig. 4b,c. The results revealed that the *V*_o_-rich Co_3_O_4_ single-unit-cell layer has a surface coverage of 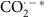
<<1, while the *V*_o_-poor Co_3_O_4_ single-unit-cell layer has a surface coverage of 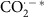
≈0.27.

**Figure 5:**
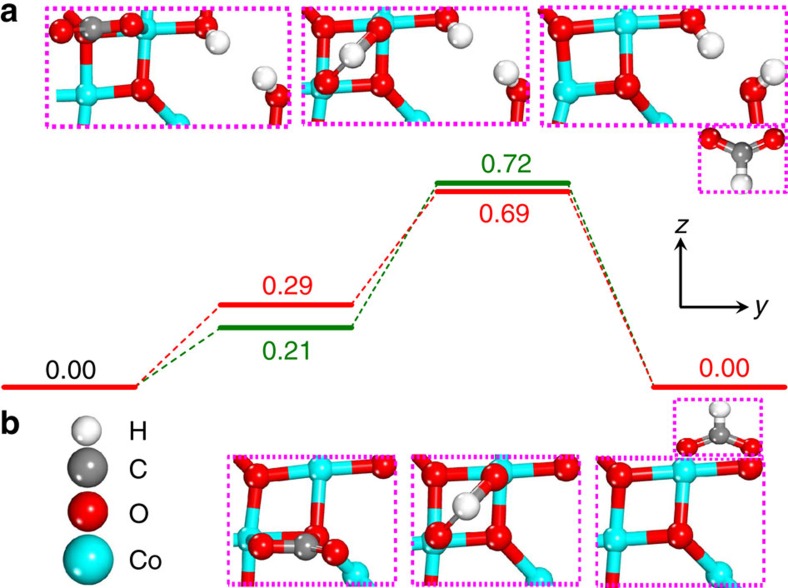
Calculated free energy diagrams. Calculated free energy diagrams for the electrochemical reduction of CO_2_ to formate on the Co_3_O_4_ single-unit-cell layers with oxygen vacancies (**a**) and the intact Co_3_O_4_ single-unit-cell layers (**b**), respectively; energy unit in eV. The first step is an electron transfer step to form 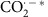 and the second step includes a simultaneous proton/electron transfer. The final state is HCOO^−^ in solvent (Solvent effect was not considered during the DFT calculations.). Values of Δ*G* are reported with at the −0.225 V versus NHE, which is the equilibrium potential of the whole reduction process. For the energy of electron is independent on pH value, so the free energy of electron is set as 0.225 V versus NHE. Asterisk represents the active site; white, red, grey and light blue spheres represent H, O, C and Co atoms, respectively.

To further verify the crucial rate-determining step, we performed density functional theory (DFT) method to calculate the full CO_2_ catalytic reduction cycle on the two materials. Based on the above XAFS, TEM and AFM results, we first optimized configurations for the model of Co_3_O_4_ single-unit-cell layers and found that Co(III) atoms rather than Co(II) atoms were exposed on the surface (Supplementary Fig. 9a,b; Supplementary Tables 1 and 2). In addition, two types of oxygen atoms were distributed on the surface of single-unit-cell layers: the one binds with three Co(III) atoms was named as O(II) and the other binds with two Co(III) atoms and one Co(II) atom was named as O(I). For the oxygen vacancies confined in Co_3_O_4_ single-unit-cell layers, the formation energy of O(II) vacancy was smaller by 0.44 eV than that of O(I) vacancy and hence it was expected that *V*_O(II)_ was the main defect in the *V*_o_-rich Co_3_O_4_ single-unit-cell layers (Supplementary Fig. 9c,d; Supplementary Table 1). Note that *V*_O(II)_ was easily recovered by hydroxyls from the dissociation of water molecule in water solution^33^ and hence the O(II) vacancy model with two hydroxyls was adopted in the following calculations for the *V*_o_-rich Co_3_O_4_ single-unit-cell layers (Supplementary Figs 10 and 11). As shown in Fig. 5, the free energy potential of each elementary step is calculated and corrected by the equilibrium potential of the whole reaction relative to the normal hydrogen electrode (NHE) (Supplementary Tables 3 and 4)^34^,^35^,^36^. To simulate the real electrochemical surroundings, extra 0.92 e^−^ or 0.62 e^−^ (Supplementary Fig. 12; Supplementary Table 5) was added in calculating the energy of 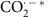 and HCOO^−^ and catalyst surfaces for *V*_o_-poor and *V*_o_-rich Co_3_O_4_ single-unit-cell layers, respectively. Although it is possible to decouple the proton and electron donations during DFT calculations, it is very difficult to separate the processes in modelling the present system. So, the energy of (H^+^+e^−^) was calculated using the computational hydrogen electrode at −0.225 V versus NHE and at this potential the energy of the extra electron is set as 0.225 eV. That is to say, at pH=6.8 of the present system, the corrected potential is −0.67 V versus SCE, which is the equilibrium potential for the CO_2_/HCOO^−^ couple^6^. At equilibrium potential, the produced HCOO^−^ has the same free energy with the reactant. Of note, in Fig. 5, the free energy of reactant CO_2_+(H^+^+e^−^)+e^−^ was set as 0.00 eV on each material, while the free energies of the 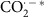, HCOO^−*^ intermediate and the product of HCOO^−^ were shifted according to it. The free energy change of the formation of 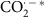 on the *V*_o_-rich Co_3_O_4_ single-unit-cell layer is higher than that on the *V*_o_-poor single-unit-cell layer, which suggests the former's lower surface coverage of 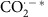 according to quasi-equilibrium assumption, fairly agreeing with the above theoretical analysis. Owing to the highest peak along the free energy surface, the formation of HCOO^−*^ intermediate binding with two Co(III) atoms through two oxygen atoms with a bidentate configuration on both surfaces (Supplementary Table 4) is the rate-limiting step of the whole reaction, which is further demonstrated by their corresponding Tafel slopes in Fig. 4b, the log(*j*_formate_) versus log([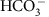]) plots in Fig. 4c as well as the corresponding theoretical analysis in Supplementary Methods. The energy barrier of the rate-limiting step is reduced by 0.11 eV and the energy barrier of the whole reaction is reduced by 0.03 eV on the *V*_o_-rich Co_3_O_4_ single-unit-cell layers than that on the *V*_o_-poor single-unit-cell layers based on the assumption that the additional barrier on top the free energy difference is almost the same for both samples. Compared with the defect free Co_3_O_4_ single-unit-cell layers, the presence of O(II) vacancy helped to stabilize the HCOO^−*^ intermediate and hence favoured the hydrogenation process. This strongly accounted for their lowered onset potential from 0.81 to 0.78 V versus SCE and decreased Tafel slope from 48 to 37 mV dec^−1^ for CO_2_ reduction into formate (Figs 3a,b and 4b), hence accelerating their overall catalytic reduction rate. In addition, the desorption process of HCOO^−^ was exothermic, indicating that the formed HCOO^−^ could be reasonably desorbed from the catalyst surfaces under reductive electrochemical conditions (Supplementary Fig. 13), which would be expected to provide enough space for sustaining the following CO_2_ reduction reactions. Furthermore, electrochemical impedance spectra in Fig. 4d revealed that the presence of O(II) vacancy led to an improved electric conductivity, which favoured enhanced charge transport in the *V*_o_-rich Co_3_O_4_ single-unit-cell layers and hence helped to promote their CO_2_ reduction activity^24^. As a consequence, theoretical and experimental results both verified that the *V*_O(II)_ confined in Co_3_O_4_ single-unit-cell layers favoured the rate-limiting H^+^ transfer step via stabilizing the HCOO^−*^ intermediate, hence lowering their overall activation energy and definitely accelerating the speed of CO_2_ reduction catalysis.

In conclusion, oxygen vacancies confined in atomic layers were put forward as an excellent platform for attaining atomic-level insights into the role of oxygen vacancies in CO_2_ reduction catalysis. *V*_o_-rich and *V*_o_-poor Co_3_O_4_ single-unit-cell layers were first controllably synthesized via a lamellar hybrid intermediate strategy and hence taken as examples to semi-quantify how oxygen vacancies matter in CO_2_ reduction. EXAFS and XPS results demonstrated the distinct oxygen vacancy concentration in these two samples, while DFT calculations revealed O(II) vacancy was the main defect in the Co_3_O_4_ single-unit-cell layers. CO_2_ adsorption isotherms revealed that the presence of O(II) vacancy facilitated CO_2_ adsorption, while DFT calculations suggested that it also favoured spontaneous HCOO^−^ desorption, which prevented catalyst deactivation. More importantly, electrokinetic results and theoretical analysis demonstrated that the donation of a proton from 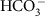 was a rate-determining step, while DFT calculations disclosed that *V*_O(II)_ confined in Co_3_O_4_ single-unit-cell layers favoured the rate-limiting proton transfer step via stabilizing the HCOO^−*^ intermediate, and hence lowered the activation energy barrier from 0.51 to 0.40 eV. This probably accelerated the speed of CO_2_ reduction, which was further confirmed by their lowered onset potential from 0.81 to 0.78 V versus SCE and decreased Tafel slope from 48 to 37 mV dec^−1^. As a result, the *V*_o_-rich Co_3_O_4_ single-unit-cell layers showed current densities of ca. 2.7 mA cm^−2^ with ca. 85% formate selectivity during the tested period of 40 h at −0.87 V versus SCE. Briefly, this work gains atomic-level insights into the role of oxygen vacancies in CO_2_ reduction catalysis through semi-quantifying the relationship among model, structure and performance, holding promise for designing efficient and robust CO_2_ reduction catalysts.

## Methods

### Synthesis of ultrathin Co(CO_3_)_0.5_(OH)·0.11H_2_O layers

In a typical procedure, 600 mg Co(acac)_3_ (Alfa Aesar) was added into a mixed solution of 60 ml ethylene glycol (Alfa Aesar) and 11 ml distilled water. After vigorous stirring for 10 min, 2.2 g CTAB (Alfa Aesar) was also added into the reacted system and then the mixture was transferred into a 100 ml Teflon-lined autoclave, sealed and heated at 180 °C for 20 h. The system was then allowed to cool down to room temperature naturally, the final product was collected by centrifuging the mixture, washed with ethanol and water for many times, and then dried in vacuum overnight for further characterization.

### Synthesis of *V*_o_-rich Co_3_O_4_ single-unit-cell layers

In a typical procedure, the as-obtained ultrathin Co(CO_3_)_0.5_(OH)·0.11H_2_O sheets were directly heated at 320 °C for 5 min in air and then cooled to room temperature. The obtained powders were collected for further characterization.

### Synthesis of *V*_o_-poor Co_3_O_4_ single-unit-cell layers

In a typical procedure, the as-obtained ultrathin Co(CO_3_)_0.5_(OH)·0.11H_2_O sheets were directly heated at 320 °C for 5 min in O_2_ and then cooled to room temperature. The obtained powders were collected for further characterization.

### Characterization

TEM images and high-resolution TEM image were performed by using a JEOL-2010 TEM with an acceleration voltage of 200 kV. X-ray diffraction patterns were recorded by using a Philips X'Pert Pro Super diffractometer with Cu Kα radiation (*λ*=1.54178 Å). XPS were acquired on an ESCALAB MKII with Mg Kα (*hυ*=1253.6 eV) as the excitation source. The binding energies obtained in the XPS spectral analysis were corrected for specimen charging by referencing C 1*s* to 284.8 eV. AFM study in the present work was performed by means of the Veeco DI Nano-scope MultiMode V system. Raman spectra were detected by a RenishawRM3000 Micro-Raman system. The Fourier transform infrared spectra were acquired on a NICOLET Fourier transform infrared spectrometer in a KBr tablets, scanning from 4,000 to 400 cm^−1^ at room temperature.

### EXAFS experimental details

An amount of 2 mg sample was homogeneously mixed with 100 mg graphite and hence pressed into circular pellets with a diameter of 10 mm for further EXAFS measurement under ambient conditions. Then, the XAFS measurements were performed at 1W1B station in BSRF. The storage rings of BSRF were operated at 2.5 GeV with the maximum current of 450 mA. Si(111) double-crystal monochromator crystals were used to monochromatize the X-ray beam. The energy resolution at Co K-edge was ∼2–3 eV. XAFS data were collected in transmission in the energy range from −130 below to 1,000 eV above the Co K-edge. The detuning was done by 30% to remove harmonics. The acquired time-dependent EXAFS data were processed according to the standard procedures using the ATHENA module implemented in the IFEFFIT software packages. The quantitative curve-fittings were carried out in the R-space with a Fourier transform k-space range of 2.8–13.8 Å^−1^ using the module ARTEMIS of IFEFFIT. The backscattering amplitude F(k) and phase shift Φ(k) were calculated using FEFF8.0 code. During the curve-fitting, the overall amplitude reduction factor *S*_0_^2^ was fixed to the best-fit value of 0.70 determined from fitting the data of bulk Co_3_O_4_. The first-nearest and second-nearest Co-O and Co-Co shells in the R-range of 1.4–3.6 Å and the k-range of 2.8–13.8 Å^−1^ were included in the fitting. Of note, during the fitting of bulk Co_3_O_4_, its coordination numbers were fixed as the nominal values (that is, the same as that of Co_3_O_4_ theory), while the internal atomic distances *R*, Debye–Waller factor *σ*^2^ and the edge-energy shift *E*^0^ were allowed to run freely. For the synthetic two samples, the structural parameters, such as the coordination number *N*, interatomic distance *R*, the Debye–Waller factor *σ*^2^ and the edge-energy shift Δ*E*_0_ were allowed to vary during the fitting process. The obtained structural parameters are summarized in Table 1. The typical curve-fitting results for the three different samples are shown in Supplementary Fig. 4.

### Electrochemical measurements

Electrochemical measurements were carried out in a three-electrode system at an electrochemical station (CHI760E). Typically, 15 mg sample and 40 μl Nafion solution (5 wt%) were dispersed in 1 ml water-ethanol solution with volume ratio of 3:1 by sonicating for 1 h to form a homogeneous ink. Then, 40 μl of the dispersion was loaded onto a glassy carbon electrode with 12 mm diameter. For CO_2_ reduction experiments, LSV with a scan rate of 20 mV s^−1^ was conducted in 60 ml CO_2_-saturated 0.1 M KHCO_3_ solution (the KHCO_3_ electrolyte was purged with CO_2_ for 30 min before the measurement.). For comparison, the LSV with a scan rate of 20 mV s^−1^ was also conducted in N_2_-saturated 0.1 M KHCO_3_ solution. The glassy carbon electrode served as the working electrode. The counter and the reference electrodes were the graphite rod and the SCE reference electrode, respectively. The outlet gases were analysed by gas chromatography (SP6800A with TDX-01 columns) equipped with thermal conductivity detector. The liquid products were quantified by nuclear magnetic resonance (Bruker AVANCE AV III 400) spectroscopy, in which 0.5 ml electrolyte was mixed with 0.1 ml D_2_O and 0.03 μl dimethyl sulfoxide (Sigma, 99.99%) was added as an internal standard. The ECSA of the working electrodes could be calculated according to the following equation: ECSA=*R*_f_*S*, where S was the real surface area of the smooth oxide electrode and *R*_f_ was the roughness factor of the working electrodes. Notably, *S* was generally equal to the geometric area of glassy carbon electrode (in this work, *S*=1.13 cm^2^). The roughness factor (*R*_f_) was estimated from the double-layer capacitance of a smooth oxide surface (60 μF cm^−2^) using the relation *R*_f_=*C*_dl_/60 μF cm^−2^. The *C*_dl_ was determined by measuring the capacitive current associated with double-layer charging from the scan rate dependence of CVs. For this, the potential window of CVs was −0.3 to −0.2 V versus SCE (0.1 M Na_2_SO_4_ solution). The scan rates were 10, 20, 50, 80, 100, 120 and 150 mV s^−1^. The *C*_dl_ was estimated by plotting the Δ*j=(j*_a_*−j*_c_) at −0.25 V versus SCE against the scan rate, in which the slope was twice that of *C*_dl_. Tafel slopes for formate production (that is, *j*_total_ × *η*_formate_) were calculated from the corresponding current densities according to the LSV curves at the current density range of 0.01–4 mA cm^−2^ and the formate Faradaic efficiency (*η*_formate_). Assuming that two electrons are needed to produce one formate ion, the Faradaic efficiency for formate production (*η*_formate_) can be calculated as follows: *η*_formate_=2*F* × *n*_formate_/*Q*=2*F* × *n*_formate_/(*I* × *t*), where *F* is the Faraday constant.

### Element analysis results

To further quantify the Co:O ratio of *V*_o_-rich and *V*_o_-poor Co_3_O_4_ single-unit-cell layers, we further perform the following two methods:

1. Inductively coupled plasma atomic emission spectroscopy: an amount of 0.1000±0.0001, g Co_3_O_4_ single-unit-cell layers were dissolved into 3 ml HCl (AR), and then 0.3 ml H_2_O_2_ (30%, AR) was added into the above solution drop by drop under stirring. After 5 min stirring, the system was heated in a sealed conical flask to totally dissolve the Co_3_O_4_, and then cooled down to room temperature naturally. Afterwards, the residual solution was transferred into 25 ml volumetric flask and then diluted with deionized water to calibration. Finally, the content of Co in Co_3_O_4_ single-unit-cell layers was measured by inductively coupled plasma atomic emission spectrometer. Five independent measurements were conducted for the same samples. The Co mass contents of *V*_o_-rich and *V*_o_-poor Co_3_O_4_ single-unit-cell layers were 0.745±0.001 and 0.737±0.001, respectively. Thus, the corresponding Co:O ratios (molar ratio) for *V*_o_-rich and *V*_o_-poor Co_3_O_4_ single-unit-cell layers were 0.793±0.001 and 0.761±0.001 according to their Co mass contents, respectively.
2. Titration: an amount of 0.1000±0.0001, g Co_3_O_4_ single-unit-cell layers were dissolved into 3 ml HCl (AR), and then 0.3 ml H_2_O_2_ (30%, AR) was added into the above solution drop by drop under stirring. After 5 min stirring, the system was heated in a sealed conical flask to totally dissolve the Co_3_O_4_, and then cooled down to room temperature naturally. Afterwards, the residual solution was transferred into 25 ml volumetric flask and then diluted with deionized water and HCl (AR) to calibration (*C*_HCl_=2 M). Then, 5 ml solution was took out and poured into a conical flask. The pH of the system was adjusted to 6 with ammonium buffer solution (pH=10). After that, 50 mg Murexide indicator was added into the mixed system and whereafter EDTA solution (0.01 M) was added drop by drop under stirring till the colour of the mixed system turned from yellow to purple. The Co mass content of *V*_o_-rich and *V*_o_-poor Co_3_O_4_ single-unit-cell layers could be obtained from the following equations: Co^2+^+EDTA→Co(EDTA)^2+^

*n*_Co_=5*C*_EDTA_*V*_EDTA_, where the *C*_EDTA_ is 0.01 M and *V*_EDTA_ is the used volume of the EDTA solution. Five independent measurements were conducted for the same samples. The volumes of the EDTA solution were 25.30±0.09 and 24.98±0.11 cm^3^ for *V*_o_-rich and *V*_o_-poor Co_3_O_4_ single-unit-cell layers, respectively. The corresponding Co molar contents for *V*_o_-rich and *V*_o_-poor Co_3_O_4_ single-unit-cell layers were 1.265±0.001 and 1.249±0.001, respectively. Thus, the corresponding Co:O ratios (molar ratio) for *V*_o_-rich and *V*_o_-poor Co_3_O_4_ single-unit-cell layers were 0.795±0.001 and 0.757±0.001, respectively.

Both the above two quantitative methods obtained the similar results of Co:O ratios in the same Co_3_O_4_ single-unit-cell layers, and, importantly, the *V*_o_-rich Co_3_O_4_ single-unit-cell layers indeed possessed higher oxygen vacancy concentration than the *V*_o_-poor Co_3_O_4_ single-unit-cell layers, which fairly agreed with the O 1*s* XPS spectra and EXAFS analysis.

### Data availability

The authors declare that the data supporting the findings of this study are available within the article and its Supplementary Information files and from the corresponding author upon reasonable request.

## Additional information

**How to cite this article:** Gao, S. *et al*. Atomic layer confined vacancies for atomic-level insights into carbon dioxide electroreduction. *Nat. Commun.* **8,** 14503 doi: 10.1038/ncomms14503 (2017).

**Publisher's note**: Springer Nature remains neutral with regard to jurisdictional claims in published maps and institutional affiliations.

## Supplementary Material

Supplementary Information Supplementary Figures, Supplementary Tables, Supplementary Methods and Supplementary References

## Figures and Tables

**Table 1:** EXAFS curve-fitting results.

**Sample**	**Path**	***N***	***R*** **(Å)**	**σ**^**2**^ **(10**^**−3**^**Å**^**2**^**)**	**ΔE**_***0***_**(eV)**
Co_3_O_4_ theory	Co-O	5.3	1.91		
	Co-Co_1_	4.0	2.85		
	Co-O_1_	5.3	3.26		
	Co-Co_2_	9.3	3.37		
	Co-O_2_	4.0	3.56		
Bulk Co_3_O_4_	Co-O	5.3	1.91±0.01	2.8±0.2	1.8±1.0
	Co-Co_1_	4.0	2.85±0.01	3.0±0.2	2.2±1.0
	Co-O_1_	5.3	3.25±0.02	5.7±0.1	1.8±1.0
	Co-Co_2_	9.3	3.37±0.01	6.0±0.2	2.2±1.0
	Co-O_2_	4.0	3.55±0.02	6.5±0.1	1.8±1.0
*V*_o_-poor Co_3_O_4_ single-unit-cell layers	Co-O	4.6±0.2	1.91±0.01	3.5±0.4	1.7±1.0
	Co-Co_1_	3.1±0.2	2.85±0.01	3.7±0.4	2.4±1.0
	Co-O_1_	4.3±0.2	3.24±0.02	7.3±0.3	1.7±1.0
	Co-Co_2_	8±0.2	3.37±0.01	8.1±0.4	2.4±1.0
	Co-O_2_	2.8±0.3	3.55±0.02	8.3±0.3	1.7±1.0
*V*_o_-rich Co_3_O_4_ single-unit-cell layers	Co-O	4.2±0.2	1.89±0.01	4.0±0.4	−2.4±1.0
	Co-Co_1_	3.1±0.2	2.84±0.01	3.9±0.4	1.9±1.0
	Co-O_1_	4.1±0.2	3.21±0.02	8.3±0.3	−2.4±1.0
	Co-Co_2_	8±0.2	3.36±0.01	8.7±0.4	1.9±1.0
	Co-O_2_	2.5±0.3	3.51±0.02	8.9±0.3	−2.4±1.0

Structural parameters around Co atoms extracted from EXAFS curve-fitting for Co_3_O_4_ theory, bulk Co_3_O_4_ fabricated according to a previous study^24^. *V*_o_-rich and *V*_o_-poor Co_3_O_4_ single-unit-cell layers. During the fitting of bulk Co_3_O_4_, its coordination numbers were fixed as the nominal values (that is, the same as that of Co_3_O_4_ theory), while the internal atomic distances *R*, Debye–Waller factor *σ*^2^, and the edge-energy shift *E*^0^ were allowed to run freely. The uncertainties in the fitting results for the samples were provided.
