# Supplementary material for: Atomic layer confined vacancies for atomic-level insights into carbon dioxide electroreduction
Source: Nat Commun. 2017 Feb 21;8:14503. doi: 10.1038/ncomms14503 (PMC5321757; doi:10.1038/ncomms14503)
Supplement: Supplementary Information — Supplementary Figures, Supplementary Tables, Supplementary Methods and Supplementary References [file ncomms14503-s1.pdf]

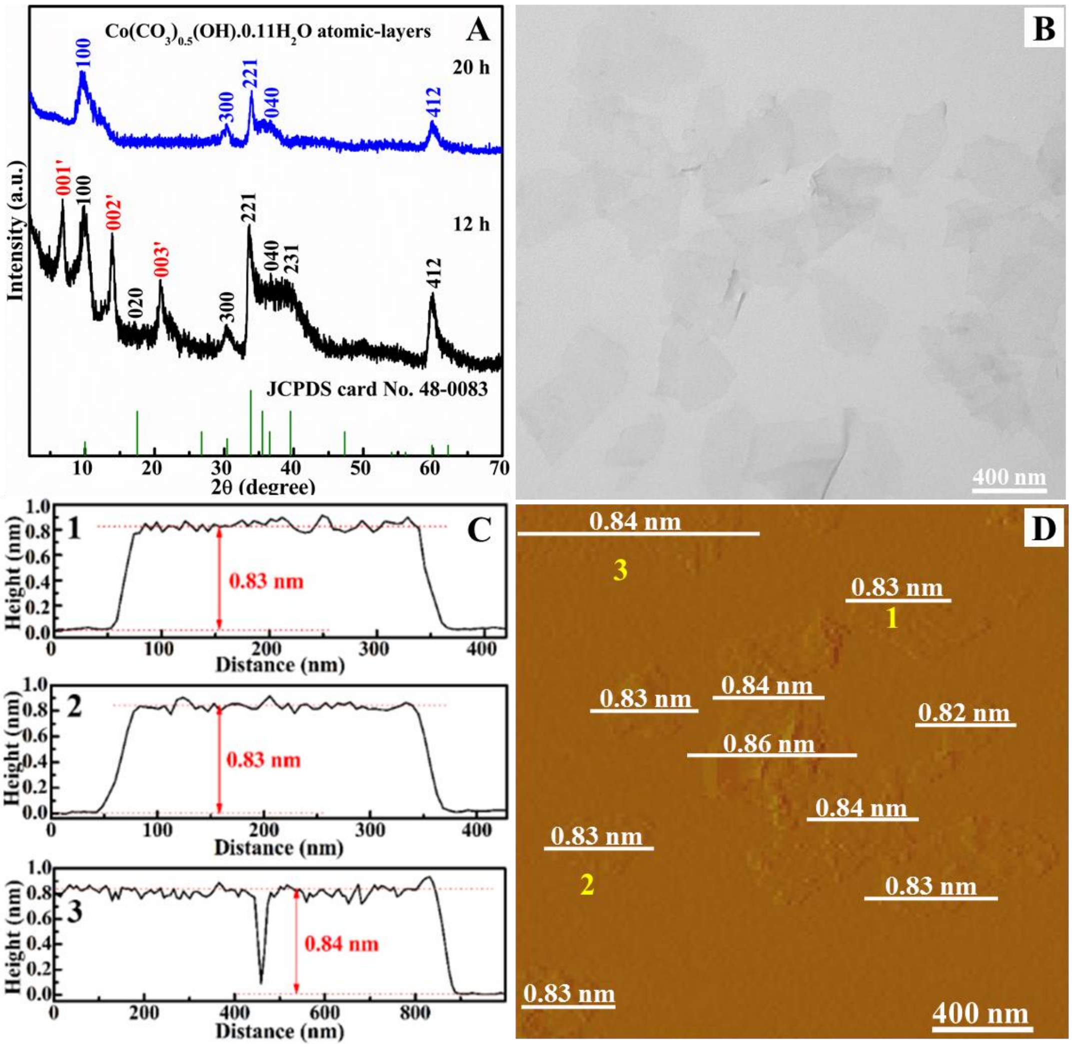

Supplementary Figure 1. Characterization for the precursors. (A) XRD patterns for the precursors obtained at different reaction time. Characterizations for the precursor of ultrathin $Co(CO_3)_{0.5}(OH)\cdot 0.11H_2O$ layers obtained at 180 °C for 20 h. (B) TEM image, (C) height profiles and (D) the corresponding AFM image; the numbers from 1 to 3 in (C) correspond to the numbers from 1 to 3 in (D).

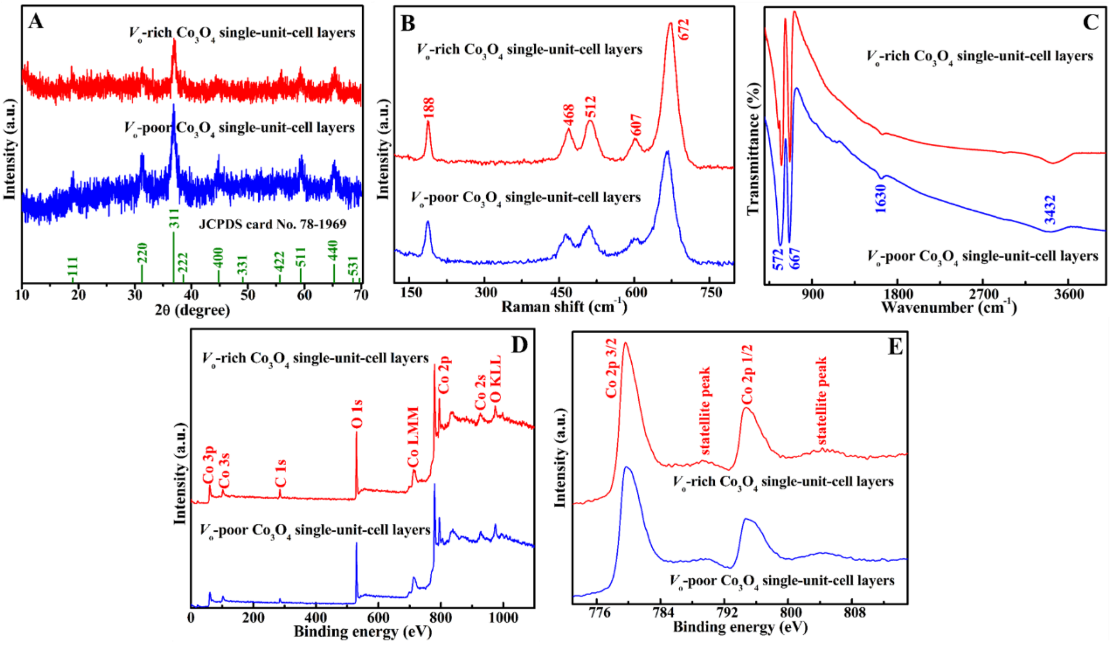

Supplementary Figure 2. Characterizations for the V_o-rich and V_o-poor Co_3O_4 single-unit-cell layers. (A) XRD patterns, (B) Raman spectra, (C) IR spectra, and (D-E) XPS spectra.

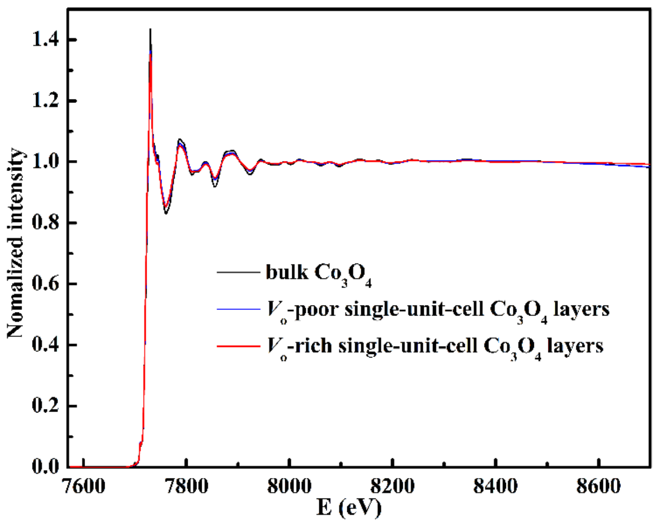

Supplementary Figure 3. **Co *K*-edge extended XAFS spectra.** Co *K*-edge extended XAFS spectra of bulk Co_3O_4, V_o-rich and V_o -poor Co_3O_4 single-unit-cell layers.

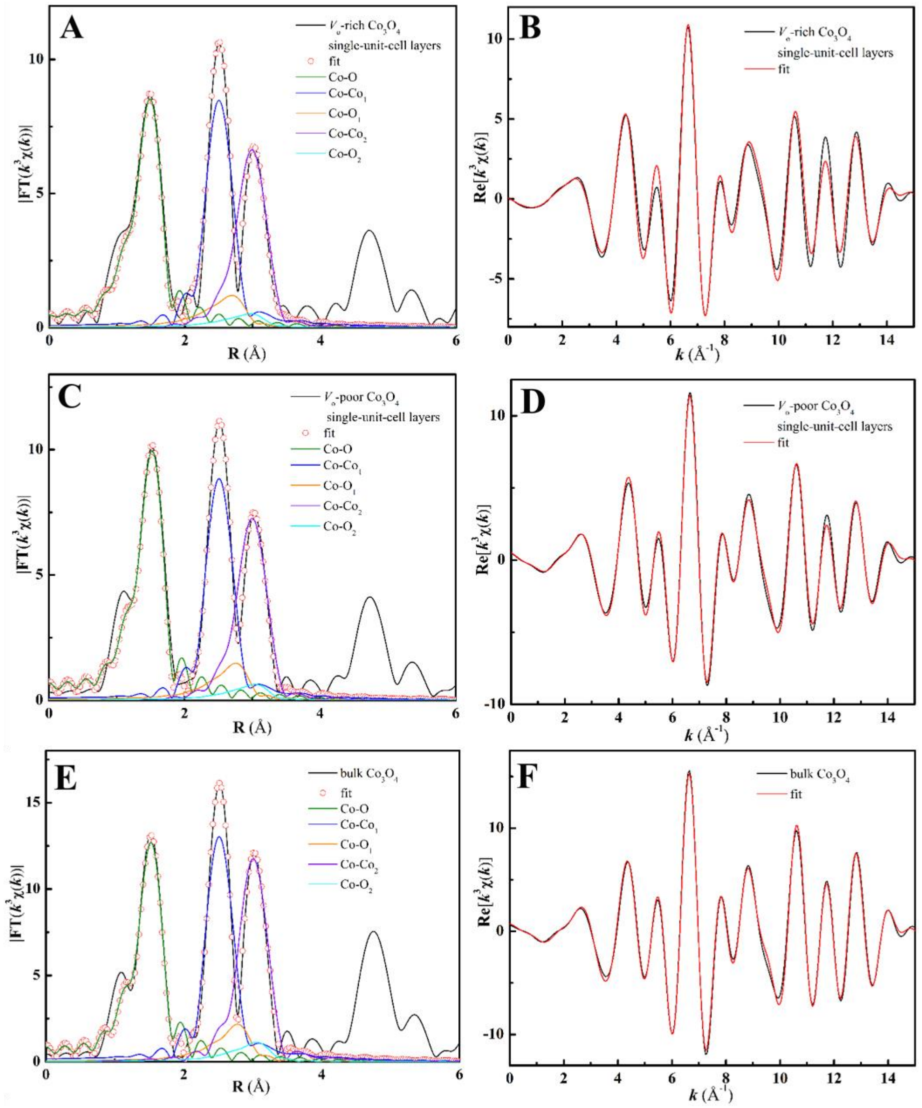

Supplementary Figure 4. Curve-fitting results. Curve-fitting results: Co *K*-edge for (A-B) V_o-rich Co_3O_4 single-unit-cell layers, (C-D) V_o-poor Co_3O_4 single-unit-cell layers, and (E-F) bulk Co_3O_4.

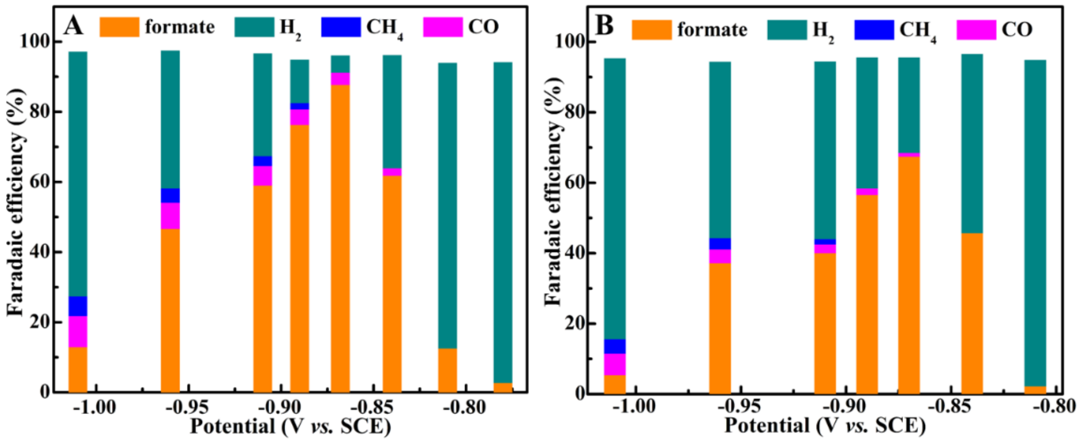

Supplementary Figure 5. Faradaic efficiencies. Faradaic efficiencies of formate, H_2, CH_4 and CO at different applied potentials for (A) V_o-rich and (B) V_o-poor Co_3O_4 single-unit-cell layers.

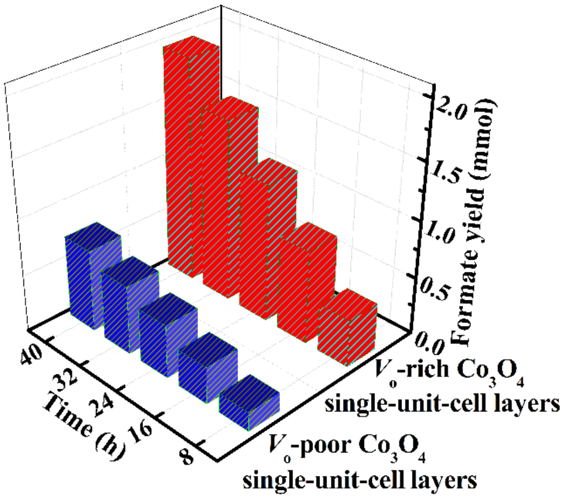

Supplementary Figure 6. **Chrono-Amperometry results.** Chrono-Amperometry results at the potential of -0.87 V vs. SCE for the V_o-rich and V_o-poor Co_3O_4 single-unit-cell layers.

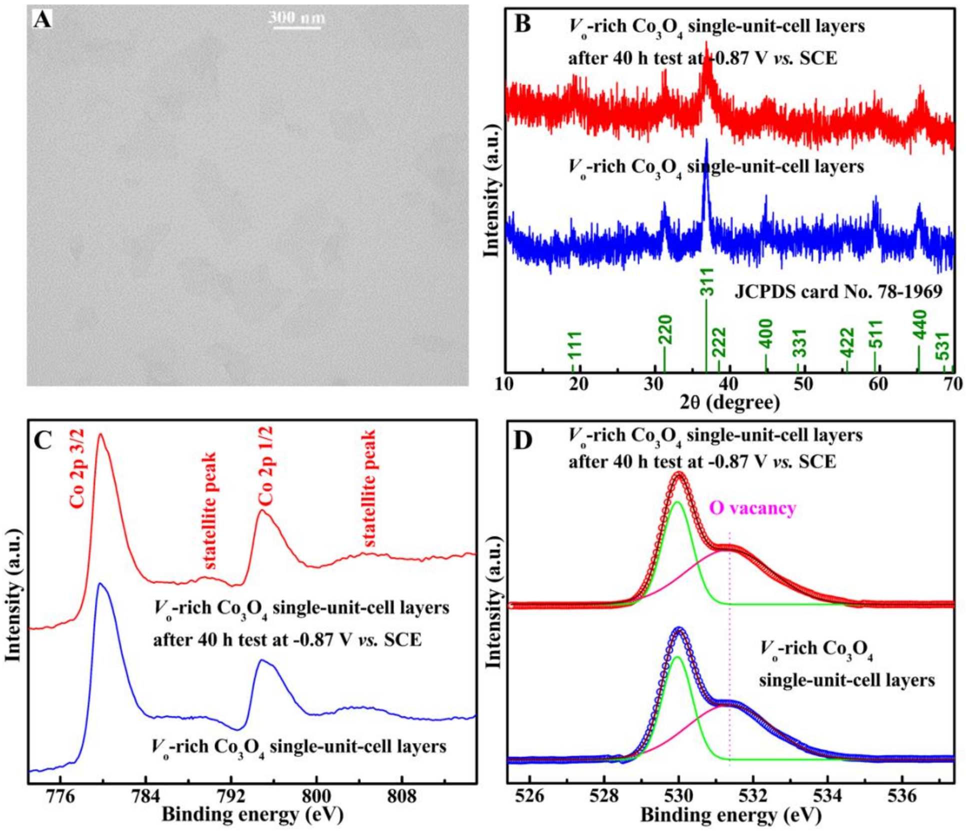

Supplementary Figure 7. **Post reaction analysis.** for (A) TEM image for the V_o-rich Co_3O_4 single-unit-cell layers after 40 h test at -0.87 V *vs*. SCE. (B) XRD pattern, (C) Co 2p spectra and (D) O 1s spectra for the V_o-rich Co_3O_4 single-unit-cell layers before and after 40 h test at -0.87 V *vs*. SCE. As revealed in the Supplementary Figure 7A, the TEM image of the V_o-rich Co_3O_4 single-unit-cell layers after 40 h test still showed the transparent sheet-like morphology, while their XRD pattern could be still indexed to cubic Co_3O_4 (Supplementary Figure 7B). In addition, their Co 2p XPS spectrum exhibited two main peaks, corresponding to the Co 2p1/2 and Co 2p3/2 spin-orbit peaks of Co_3O_4 (Supplementary Figure 7C). Thus, the above TEM image, XRD pattern and XPS spectrum strongly demonstrated that the morphology and phase of the V_o-rich Co_3O_4 single-unit-cell layers do not have any obvious variation after 40 h electroreduction test. To investigate the stability of oxygen vacancy concentration throughout the long term test, O 1s XPS spectrum was also performed on the V_o-rich Co_3O_4 single-unit-cell layers after 40 h test. As showed in the Supplementary Figure 7D, one can clearly see that the O 1s spectrum did not show any obvious variation relative to that of the V_o-rich Co_3O_4 single-unit-cell layers before 40 h test, which implied the strong stability of oxygen vacancies during electroreduction test.

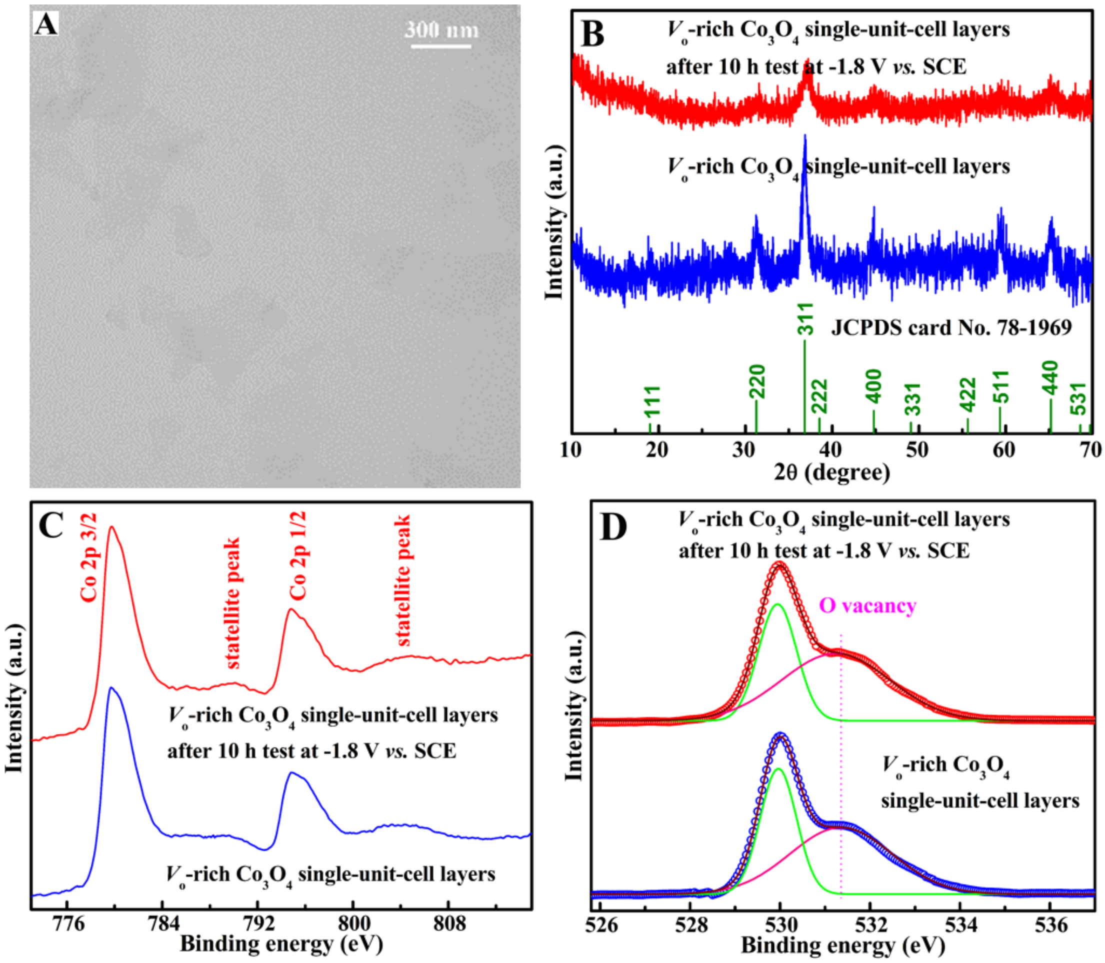

Supplementary Figure 8. **Post reaction analysis.** (A) TEM image for the V_o-rich Co_3O_4 single-unit-cell layers after 10 h test at -1.8 V *vs*. SCE. (B) XRD pattern, (C) Co 2p spectra and (D) O 1s spectra for the V_o-rich Co_3O_4 single-unit-cell layers before and after 10 h test at -1.8 V *vs*. SCE. To further demonstrate the stability of V_o-rich Co_3O_4 single-unit-cell layers under reducing conditions, we conducted the electroreduction test at a very negative potential of -1.8 V *vs*. SCE for 10 h and hence fully characterized their morphology and phase. As shown by TEM image, XRD pattern and XPS spectra in the Supplementary Figure 8, the morphology and phase as well as the oxygen vacancy concentration do not encounter any obvious change after the 10 h test at -1.8 V *vs*. SCE, further verifying the superior stability of the V_o-rich Co_3O_4 single-unit-cell layers.

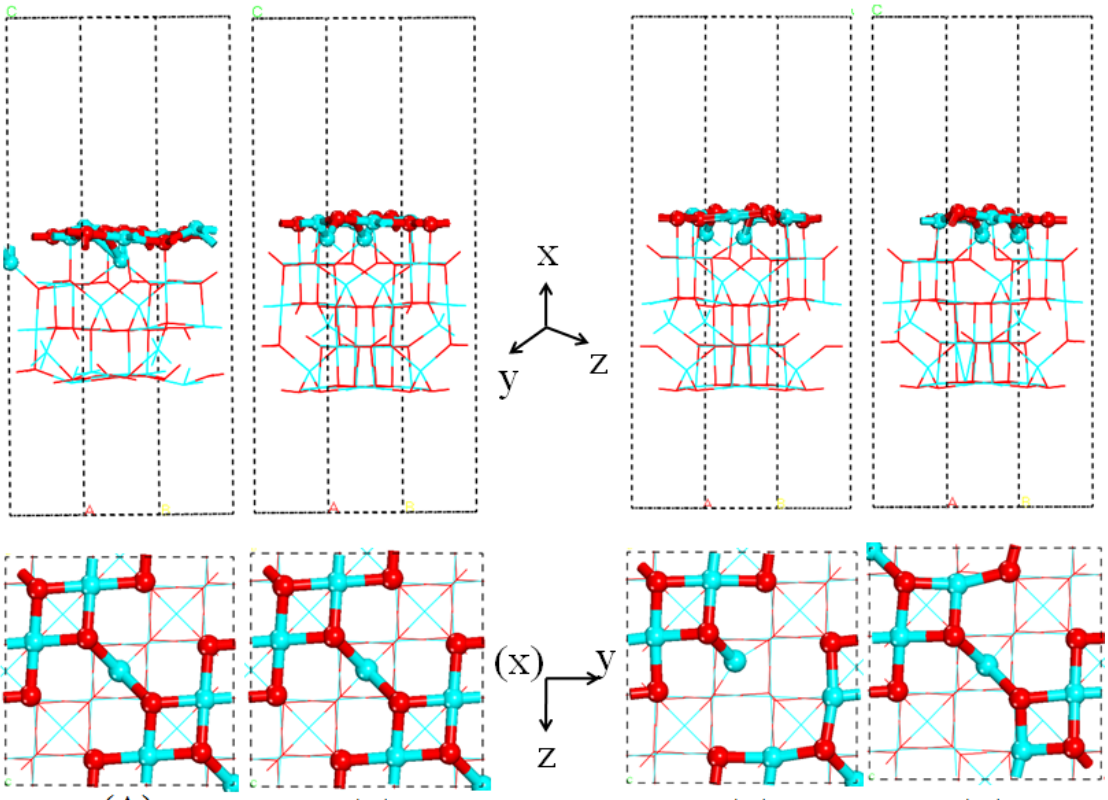

Supplementary Figure 9. **Optimized structure.** Optimized structure of Co_3O_4 single-unit-cell layers containing Co(II) atoms named Co(II)-Co_3O_4 single-unit-cell layers (A), only Co(III) atoms named as Co(III)-Co_3O_4 single-unit-cell layers (B), $V_{O(I)}$(O(I) linked with Co(II) atom) on the Co(III) surface named as Co(III)-$V_{O(I)}$-Co_3O_4 single-unit-cell layers (C) and $V_{O(II)}$ (O(II) only linked with Co(III) atoms) on the Co(III) surface named as Co(III)-$V_{O(II)}$- Co_3O_4 single-unit-cell layers (D). O atom in red, Co atom in cyan. The coordinate of visual angle is in the middle.

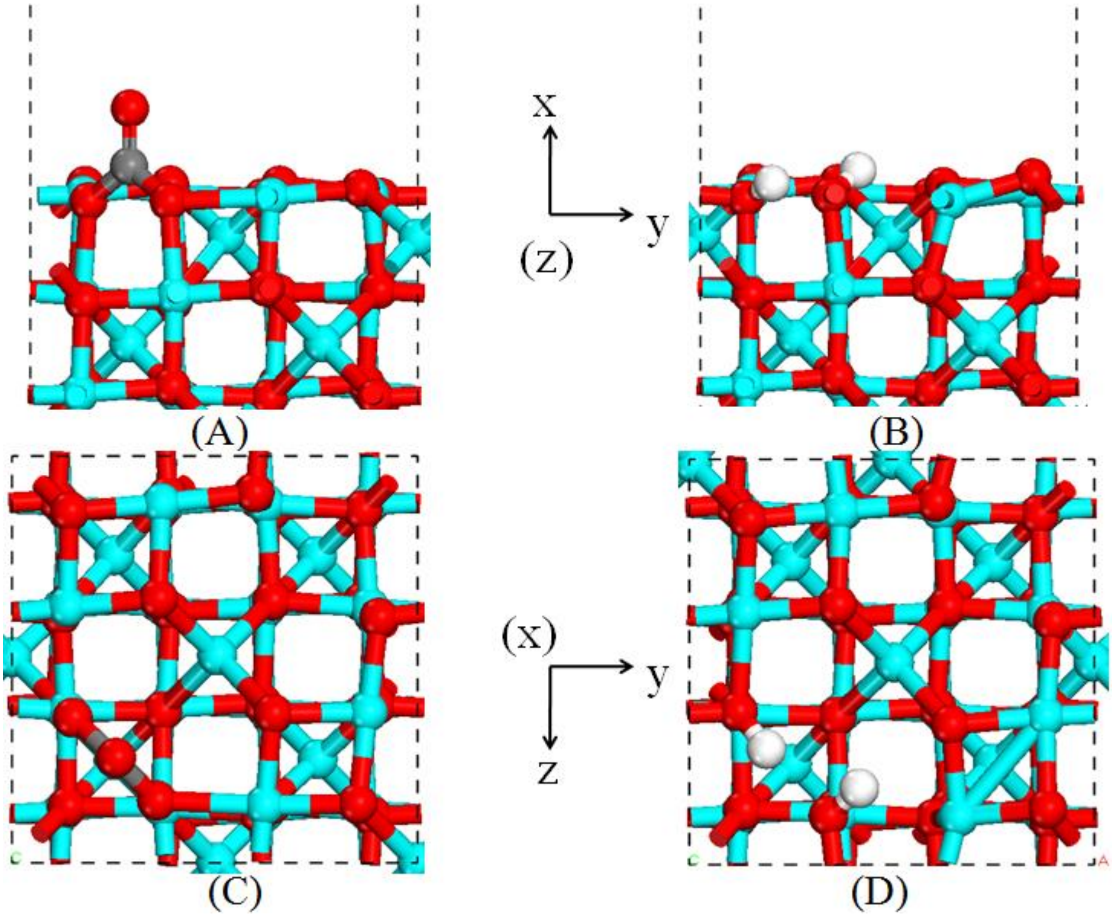

Supplementary Figure 10. **CO_2 and H_2O adsorption.** (A, C) CO_2 and (B, D) H_2O adsorption on the O(II) vacancy of Co_3O_4 single-unit-cell layers, O atom in red, C atom in grey, Co atom in cyan, H atom in white. At $V_{O(II)}$ site, the adsorption energy of H_2O with -1.45 eV is lower than CO_2 by 0.94 eV, which indicates that water molecules preferentially occupy $V_{O(II)}$ site than CO_2. The coordinate of visual angle is in the middle.

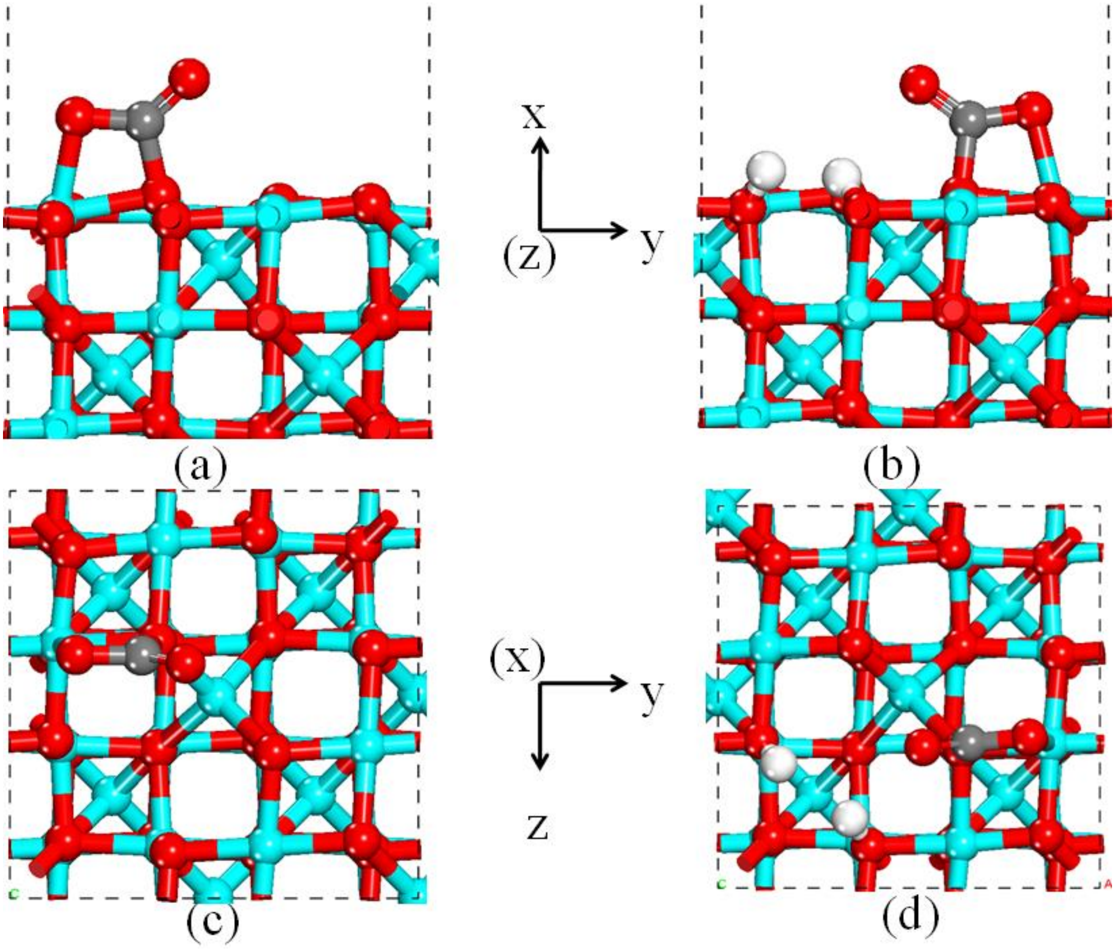

Supplementary Figure 11. **CO_2 adsorption.** CO_2 adsorption on the Co(III)-Co_3O_4 single-unit-cell layers (A, C) and Co(III)-$V_{O(II)}$-Co_3O_4 single-unit-cell layers H*-O*H in the charged system (B, D), O atom in red, C atom in grey, Co atom in cyan, H atom in white. The coordinate of visual angle is in the middle.

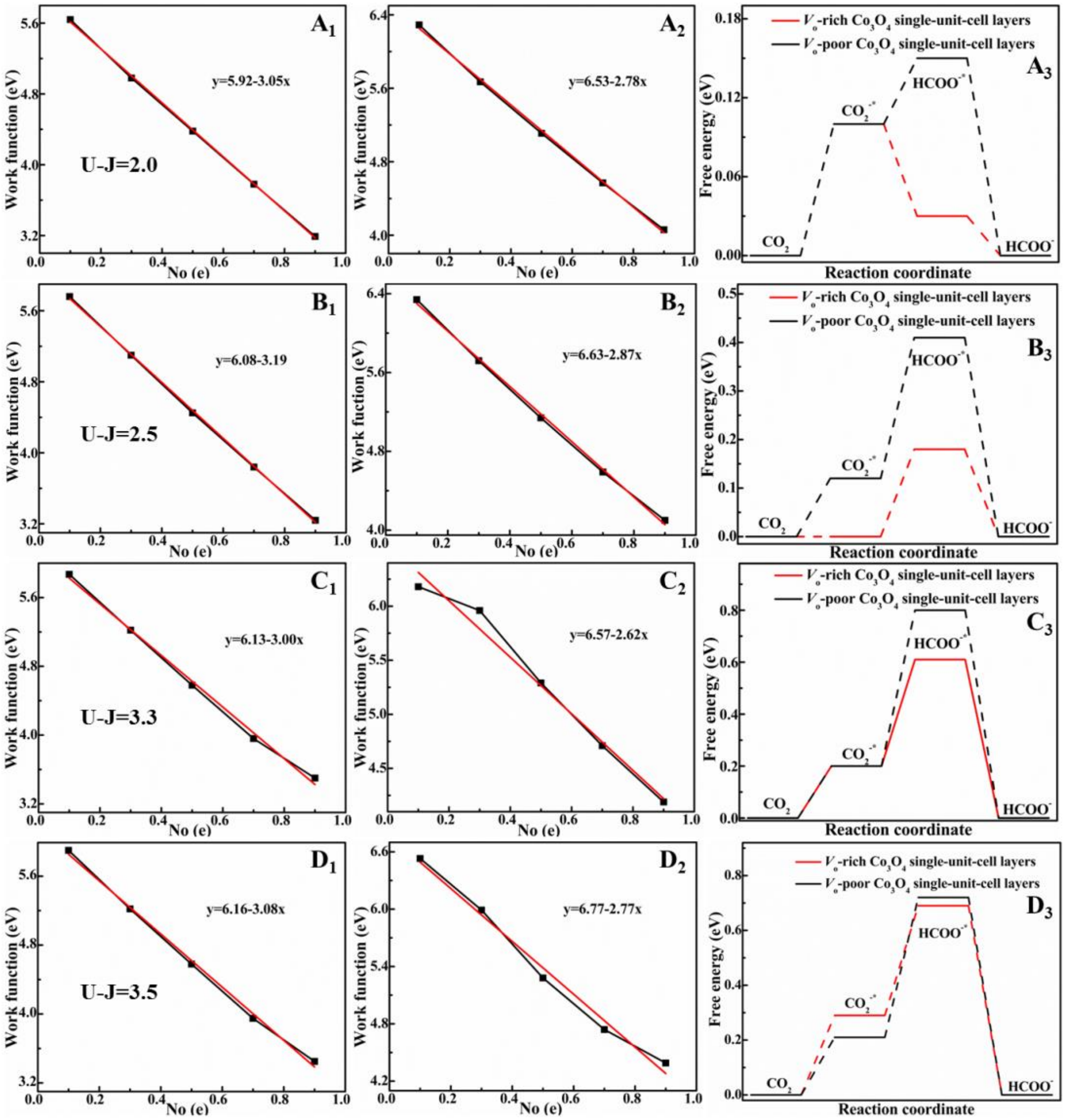

Supplementary Figure 12. Relationships between work function and electron number. The relationships between work function and electron number of (A_1-D_1) V_o-rich and (A_2-D_2) V_o-poor Co_3O_4 single-unit-cell layers system in different U-J value. (A_3-D_3) The corresponding comparison of free energy diagram in different U-J value for V_o-rich and V_o-poor Co_3O_4 single-unit-cell layers system. The values of U-J are 2.0, 2.5, 3.3, and 3.5 for Figure 13A-D， respectively.

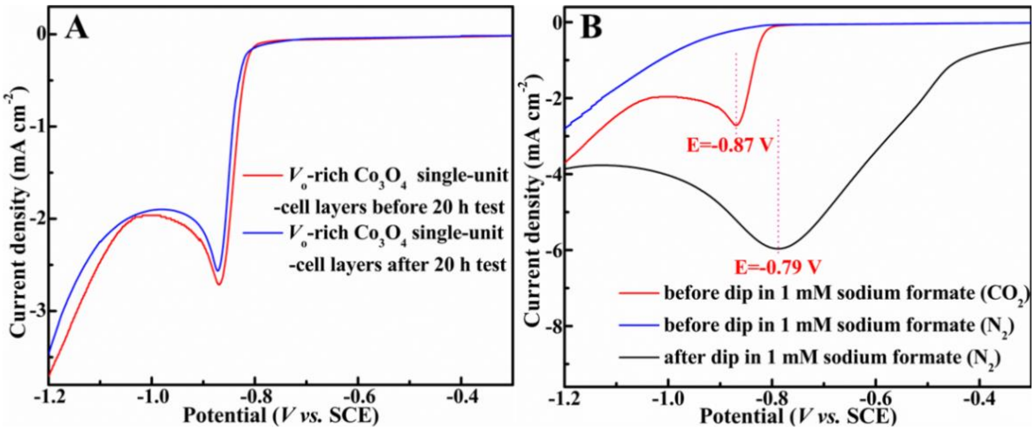

Supplementary Figure 13. Linear sweep voltammetric curves. Linear sweep voltammetric curves of V_o-rich Co_3O_4 single-unit-cell layers in a CO_2 saturated- and N_2 saturated- 0.1 M $KHCO_3$ aqueous solution. (A) Linear sweep voltammetric curves before and after 20 h measurement at -0.87 V *vs.* SCE. (B) Linear sweep voltammetric curves before and after dip in 1 mM sodium formate.

Supplementary Table 1. Total energy. The total energy of Co(II)-Co_3O_4 single-unit-cell layers (Co(II)), Co(III)-Co_3O_4 single-unit-cell layers (Co(III)), Co(III)-$V_{O(I)}$-Co_3O_4 single-unit-cell layers (Co(III)-$V_{O(I)}$), Co(II)-$V_{O(II)}$-Co_3O_4 single-unit-cell layers (Co(III)-$V_{O(II)}$).

	E/eV
Co(II)	-340.262
Co(III)	-401.242
Co(III)-$V_{O(I)}$	-394.488
Co(III)-$V_{O(II)}$	-394.930

Supplementary Table 2. Surface energy. The calculated surface energy of Co(II)-Co_3O_4 single-unit-cell layers (Co(II)) and Co(III)-Co_3O_4 single-unit-cell layers (Co(III)) under the Co-rich and O-rich condition.

	M_{Co}/eV	μ_O/eV	Co(II)/(J/m^2)	Co(III)/(J/m^2)
Co-rich	-4.34	-7.33	0.87	1.64
O-rich	-7.54	-4.93	1.65	0.86

Supplementary Table 3. **Total energies of CO_2 and H_2.** Total energies (E) of CO_2 and H_2 from DFT and ZPE and TS contributions to the free energies at the standard conditions.

Species	E	ZPE	TS
CO_2	-22.98	0.32	0.66
H_2	-6.76	0.28	0.44
$HCOO^-$	-	0.55	0.74
C*O*O	-	0.35	0.15
HCO*O*	-	0.65	0.14

Supplementary Table 4. **Frequencies.** Frequencies of adsorbed species.

Adsorbed species	Frequency (cm^{-1})
C*O*O	1837.16, 1149.47, 724.18, 719.35, 487.41, 339.22, 213.63, 166.01, 50.71
HCO*O*	2923.58, 1542.37, 1343.62, 1295.40, 950.96, 761.03, 403.80, 368.64, 331.21, 217.43, 171.95, 133.26

Supplementary Table 5. **Free energy changes**. The electrons need (N) to be added to the slab models and the free energy changes (ΔG) of each elementary step according to different U_{eff} values. The formation energy (E_f) of V_o is calculated as $E_f=E_{Ov}+1/2O_2(gas)-E_{perf}$.

		U_{eff}	***2.0***	***2.5***	***3.3***	***3.5***
E_f /eV			*1.75*	*1.56*	*1.14*	*1.23*
N/e		*V_o-poor*	*0.83*	*0.84*	*0.89*	*0.92*
		V_o-rich	*0.54*	*0.57*	*0.62*	*0.62*
ΔG/eV	$CO_2(g) + e + * \rightarrow CO_2^{\cdot-}*$	*V_o-poor*	*0.10*	*0.12*	*0.20*	*0.21*
		V_o-rich	*0.10*	*0.00*	*0.20*	*0.29*
	$CO_2^{\cdot-}* + H^+ + e \rightarrow HCOO^-*$	*V_o-poor*	*0.05*	*0.29*	*0.60*	*0.51*
		V_o-rich	*-0.07*	*0.18*	*0.41*	*0.40*

Supplementary Methods

Computational details and models: All the calculations were calculated based on spin-polarized periodic density functional theory method (DFT) implemented in Vienna ab initio simulation package (VASP).[1,2] To consider the strong correlated electronic states in cobalt oxide, U-J = 3.5 was set to describe the system, which has been adopted in previous theoretical work.[3]

The electron-ion interactions were described by the projector augmented wave (PAW) method[4] and the electron exchange and correlation energy were treated with the gradient corrected Perdew-Burke-Ernzerh of (GGA-PBE)[5] functional. The kinetic cutoff energy for plane-wave basis set was set as 400 eV. For geometric optimization, the total energy convergence was set to be smaller than 10^{-5} eV, and the force convergence was set to be lower than 0.02 eV/Å. Electron smearing of σ=0.01 eV was used following the Methfessel-Paxton scheme.[6] Brillouin zone sampling was employed using a Monkhorst-Packing grid 5×5×1.[7] For technique reason, dipole correction is not included in our calculations. We have to point that for test calculations, only 3×3×1 Monkhorst-Packing grid.

The adsorption energy was defined as $E_{ads}=E_{A/ns}-[E_{ns}+E_{A}]$; where $E_{A/ns}$ and E_{ns} is the total energy of the Co_3O_4 single-unit-cell layers with and without adsorbate A, and E_A was the energy of gas phase molecule.

Firstly, two symmetric models along Co_3O_4 (100) crystal plane were built to compare with experimental structure. One was Co(II)-exposed nanosheet named as Co(II)-Co_3O_4 single-unit-cell layers and the other was Co(III)-exposed nanosheet named as Co(III)-Co_3O_4 single-unit-cell layers. We adopted ($\sqrt{2}\times\sqrt{2}$) surface supercell, five atom layers with the orientation of the (100) facet (one Co(III)-O atom layer and Co(II) atoms close to Co(III)-O layer named as an atom layer, and the fifth atom layer only had the Co(III)-O atom layer) and all atoms were relaxed.

In order to verify the exact configuration in experiments, the surface energy of two models were calculated (Supplementary Table 1). The surface energy of Co_3O_4 single-unit-cell layers was calculated as:

$$E_{surf} = \frac{1}{2A}(E_{total} - n\mu_{Co} - m\mu_{O}) \quad (1)$$

where E_{total} was the total energy of Co(II)-Co_3O_4 single-unit-cell layers or Co(III)-Co_3O_4 single-unit-cell layers, μ_{Co} and μ_{O} were the chemical potential of Co and O atom, respectively. n and m were the number of Co and O atom. The surface energy depended on the preparation conditions such as Co-rich or O-rich. Under Co-rich condition, μ_{Co} was assumed as the energy of bulk Co, while the chemical potential of O atom could be calculated by the following thermodynamics equilibrium relation:

$$3\mu_{Co} + 4\mu_{O} = \mu_{Co_3O_4} \quad (2)$$

Under the O-rich condition, μ_{O} was obtained from the ground-state of O_2 molecule, that was, $\mu_O = 1/2\mu_{O_2}$, and the chemical potential of Co was calculated by the above Eq. 2.

Under Co-rich condition, the surface energy of Co(II) with 0.87 J/m^2 was smaller than Co(III) by 0.77 J/m^2 on the Supplementary Table 2. But in oxygen atmosphere, the surface energy of Co(III) with 0.86 J/m^2 was smaller than Co(II) by 0.79 J/m^2, which indicated that Co(III) was more stable than Co(II) in oxygen atmosphere. Furthermore, it was noticed that the thickness of optimized nanosheet was 8.50 Å for Co(III) and 7.3 Å for Co(II). So for geometric point of view, the thickness of Co(III) was well consistent with experimental data of 8.4 Å. Thus, Co(III) model was adopted for all below calculations.

To simulate the V_o-rich Co_3O_4 single-unit-cell layers, oxygen vacancies were created on the Co(III) surface. There were two types of oxygen atoms on Co(III) named as O(I) and O(II). O(I) connects with one Co(II) atom and two Co(III) atoms, while O(II) connected with three Co(III) atoms. The total energy of O(II) vacancy ($V_{\text{O(II)}}$) was 0.44 eV lower than that of O(I) vacancy ($V_{\text{O(I)}}$) as shown in Supplementary Table 1, which indicated that $V_{\text{O(II)}}$ was more stable than $V_{\text{O(I)}}$. Thus it was expected that $V_{\text{O(II)}}$ was the main defect on the V_o-rich Co_3O_4 single-unit-cell layers. At $V_{\text{O(II)}}$ site, the adsorption energy of H_2O with -1.45 eV was lower than CO_2 by 0.94 eV, which indicated that water molecules preferentially occupied $V_{\text{O(II)}}$ site

than CO_2, which also agreed well with the case on other metal oxides, such as TiO_2.[8]

Computational hydrogen electrode: A reaction pathway on CO_2 to $HCOO^-$ was analyzed in the current work. The free energy change of this step involved an electrochemical proton-electron transfer, which would be a function of applied electrode potential. The effect of applying this potential was based upon a technique, described earlier by Nørskov et al.,[9] which we were herein referring to as the computational hydrogen electrode (CHE) model.

In this technique, zero voltage was defined based on the normal hydrogen electrode (NHE), in which the reaction

$$H^+ + e^- \leftrightarrow \frac{1}{2}H_2 \quad (3)$$

was defined to be in equilibrium at zero voltage relative to NHE. Therefore, in the CHE, the chemical potential of a proton-electron pair, $\mu(H^+)+\mu(e^-)$ was defined as $G[H^+ + e^-] = \frac{1}{2}G[H_2] - eU$, where U is the electrochemical potential relative to NHE.

As an example, $COO^{\cdot-*} + H^+ + e^- \rightarrow HCOO^{-*}$ (4)

where an asterisk (*) indicated that the species were adsorbed on the surface of Co_3O_4 single-unit-cell layers, the free energy change of the above reaction would thus be:

$$\Delta G = \mu_{HCOO^{-*}} - \mu_{COO^{\cdot-*}} - [\mu_{H+} + \mu_{e-}] = \mu_{HCOO^{-*}} - \mu_{COO^{\cdot-*}} - 1/2\mu_{H2} \quad (5)$$

where the chemical potential of $HCOO^{-*}$ and $COO^{\cdot-*}$ adsorbed species were calculated by standard DFT techniques (*ΔE*) after Zero point energy(*ZPE*) and entropy(*TS*) corrections in Supplementary Table 3.

$$\Delta G = \Delta E + \Delta ZPE - T\Delta S \qquad (6)$$

where *T* is the temperature, *ΔS* is the entropy change. For the *ZPE*, the vibrational frequencies of adsorbed species (C*O*O, HCO*O*) were calculated to obtain *ZPE* contributions in the free energy expression, among $ZPE = \sum_i \frac{1}{2} h\nu_i$, i is the frequency number, ν_i is the frequency with unit cm^{-1} as listed in Supplementary Table 4. Moreover the entropies of free gases were just considered from the *NIST* database, and the adsorbed species were only taken vibrational entropy (S_v) into account, as showed in the following formula 7:

$$S_V = \sum_i R\left\{\frac{hv_i}{k_BT}\left[\exp\left(\frac{hv_i}{k_BT}\right)-1\right]^{-1} - \ln\left[1-\exp\left(-\frac{hv_i}{k_BT}\right)\right]\right\} \quad (7)$$

among which R=8.314 J mol^{-1} K^{-1}, T=298.15 K, h=6.63*10^{-34} J·s, k_B=1.38*10^{-23} J K^{-1}, i is the frequency number，ν_i is the vibrational frequency (unit is cm^{-1}). So the contribution of free energy for vibrational entropy (TS) about adsorbed CO_2* and HCOO* species is calculated as 0.15 and 0.14 eV, respectively. As derived in theoretical analysis below, the rate-limiting step is the attachment of the H and the most abundant surface species that is adsorbed CO_2. Thus the CO_2 adsorbed surface is used to evaluate how much additional electron should be added to represent the applied potential. At applied potential, the overpotential (η) of the V_o-poor and V_o-rich Co_3O_4 single-unit-cell layers are 0.14 and 0.11 V, respectively.

In order to simulate the real electrochemical surroundings, the work function (Φ) of each material should be tuned to get the proper overpotential according to the definition of absolute electrochemical potential.[10]

$$U/V= (\Phi - 4.60)/eV,\ \eta = -0.225 - U$$

$$\Phi/eV = 4.60/eV + (-0.225 - \eta)/V$$

where Φ is the work function relative to reference level, 4.60 eV is introduced to account for the work function of normal hydrogen electrode (NHE), η is overpotential.[11,12] So the work function of V_o-poor and V_o-rich Co_3O_4 single-unit-cell layers are adjusted to be 4.23 and 4.26 eV, respectively. The work functions were adjusted by inserting electrons, as illustrated in Supplementary Figure 12D_1 and D_2. So the electrons should be added to 0.92 e^- and 0.62 e^- for V_o-poor and V_o-rich Co_3O_4 single-unit-cell layers.

According to Brønsted-Evans-Polanyi[13,14] relation, there was a linear relationship between the activation energy and the reaction energy for an elementary reaction, thus it was expected that the activation energy on the V_o-rich Co_3O_4 single-unit-cell layers was lower than that of perfect Co_3O_4 single-unit-cell layers. Thus, from reaction energy point of view, the V_o-rich Co_3O_4 single-unit-cell layers surface was more active for the reduction of CO_2.

Supplementary theoretical analysis:

1.Important questions arise: is HCO_3^- the sole proton source? Does the proton source come from H_2O?

1.1 If the proton donor was HCO_3^-, the reaction pathway in the presence system can be expressed as follows:

$$CO_2 + e^- + * \rightarrow CO_2^{\cdot -*} \quad (8)$$

$$CO_2^{\cdot -*} + HCO_3^- + e^- \rightarrow HCOO^{-*} + CO_3^{2-} \quad (9)$$

$$HCOO^{-*} \rightarrow HCOO^- + * \quad (10)$$

where the * denotes a catalytically active site.

If a one-electron rate-limiting process (8) is assumed, then the partial current of $HCOO^-$ can be described as follows[15,16]:

$$i_{HCOO^-} = nFk_{(8)}P_{CO_2}(1-\theta)\exp\left(\frac{-\beta_{(8)}EF}{RT}\right) \quad (11)$$

where θ is the total surface coverage, E is the applied potential, P_{CO_2} is the partial pressure of CO_2, $k_{(8)}$ is a rate constant for reaction (8), β is the symmetry factor and is assumed to be equal to 0.5, F is Faraday's constant, R is the gas constant, and T is the temperature.

With an assumption of low surface coverage, the derived value of the Tafel slope ($\partial E/\partial\, log i_{HCOO^-}$) is 2.3RT/βF, or 118 mV dec^{-1}. The expected Tafel slope of around 118 mV dec^{-1} is consistent with the slope of the bulk Co_3O_4 electrode, suggesting that the one electron process represented in reaction (8) is rate limiting for CO_2 reduction on bulk Co_3O_4 surfaces.[17]

In sharp contrast, Co_3O_4 single-unit-cell layer shows a clear decrease in the Tafel slope to approximately 40 mV dec^{-1}, indicating a fast initial electron transfer on the Co_3O_4 single-unit-cell layer.[18-22] Following this fast electron donation step is likely a rate limiting proton donation from HCO_3^-:

$$CO_2^{\cdot -*} + HCO_3^- + e^- \rightarrow HCOO^{-*} + CO_3^{2-}$$

On the basis of this hypothesis, our rate expression can be rewritten to be dependent on the surface coverage of the $CO_2^{\cdot -*}$ intermediate:

$$i_{HCOO^-} = nFk_{(9)}\theta_{CO_2^{\cdot -*}}[HCO_3^-] \quad (12)$$

where $\theta_{CO_2^{\cdot -*}}$ is the surface coverage of $CO_2^{\cdot -*}$ intermediate.

If we assume that the first step in the mechanism is in fast equilibrium, then we can write according to Equation 1:

$$k_{(8)} P_{CO_2} (1-\theta) \exp\left(\frac{-\beta_{A1} EF}{RT}\right) = k_{-(8)} \theta_{CO_2^{\cdot -*}} \exp\left(\frac{(1-\beta_{(8)})EF}{RT}\right)$$

$$\frac{\theta_{CO_2^{\cdot -*}}}{1-\theta} = K_{(8)} P_{CO_2} \exp\left(\frac{-EF}{RT}\right)$$

Assuming θ (the overall coverage) is small yields Equation 13:

$$i_{HCOO^-} = nFK_{(8)} k_{(9)} P_{CO_2} [HCO_3^-] \exp\left(\frac{-EF}{RT}\right) \quad (13)$$

To show the reaction order with respect to these variables, we first take the log of both sides of Equation 13 to obtain:

$$\log(i_{HCOO^-}) = \log\left(\mathrm{nF} K_{(8)} k_{(9)} P_{CO_2}\right) + \frac{-EF}{2.3RT} + \log([HCO_3^-]) \quad (14)$$

The applied potential E is made up of two components: the reversible potential of the reaction and the applied overpotential that we hold constant in these experiments. According to the overall reaction:

$$CO_2 + HCO_3^- + 2e^- \rightarrow HCOO^- + CO_3^{2-} \quad (15)$$

the Nernst equation could be written as:

$$\mathrm{E} = E^{rev} - \eta \quad (16)$$

Substituting in Equation 4 yields:

$$\log(i_{HCOO^-}) = \log\left(\mathrm{nF} K_{(8)} k_{(9)} P_{CO_2}\right) - \frac{FE^{rev}}{2.3RT} + \frac{F\eta}{2.3RT} + \log([HCO_3^-])$$

The reversible potential can be related in terms of the Nernst equation (Equation 17). This expression takes into account an additional factor: changes in chemical potential due to the varying bicarbonate concentration. Thus, we can write:

$$E^{rev} = E^{\theta} - \frac{2.3RT}{2F} \log\left(\frac{[HCOO^-]\,[CO_3^{2-}]}{P_{CO_2}\,[HCO_3^-]}\right) \quad (17)$$

Here, the dissociation of HCO_3^- is taken into account since the electrolyte is 0.1 M $KHCO_3$:

$$HCO_3^- \rightarrow H^+ + CO_3^{2-}$$

$$\frac{[CO_3^{2-}]}{[HCO_3^-]} = \frac{K_{HCO_3^-}}{H^+}$$

With these substitutions into the overall rate law, the partial derivative of the $HCOO^-$ current density with respect to bicarbonate concentration at a constant overpotential will yields an expression for our reaction order equal to 1, as shown below:

$$\log(i_{HCOO^-}) = \log\left(\mathrm{nF}K_{(8)}k_{(9)}P_{CO_2}\right) - \frac{FE^{\theta}}{2.3RT} + \frac{F\eta}{2.3RT} + \log([HCO_3^-]) + \frac{1}{2}\log(\frac{[HCOO^-]\,[CO_3^{2-}]}{P_{CO_2}\,[HCO_3^-]})$$

$$\log(i_{HCOO^-}) = \log\left(\mathrm{nF}K_{(8)}k_{(9)}P_{CO_2}\right) - \frac{FE^{\theta}}{2.3RT} + \frac{F\eta}{2.3RT} + \log([HCO_3^-]) + \frac{1}{2}\log(\frac{[HCOO^-]}{P_{CO_2}}) + \frac{1}{2}\log(\frac{K_{HCO_3^-}}{H^+})$$

Therefore, $\frac{\partial \log(i_{HCOO^-})}{\partial [HCO_3^-]} = 1$, which means first-order dependence of the reaction rate on the concentration of HCO_3^-. This theoretical result fairly consists with our experimental result in Figure 4c, further confirming that the H^+ donation from HCO_3^- was the rate-limiting step for both the V_o-rich and V_o-poor Co_3O_4 single-unit-cell layers.

1.2 If the proton donor was H_2O, then the overall reaction would change to $CO_2 + H_2O + 2e^- \rightarrow HCOO^- + OH^-$ and the Nernst equation would be:

$$E^{rev} = E^{\theta} - \frac{2.3RT}{2F}\log(\frac{[HCOO^-][OH^-]}{P_{CO_2}\,[H_2O]})$$

For Co_3O_4 single-unit-cell layer, the rate expression becomes:

$$i_{HCOO^-} = nFK_{(8)}k_{(9)}P_{CO_2}[H_2O]\exp\left(\frac{-EF}{RT}\right)$$

While we obtain the expected values for the Tafel slope, there would be no explicit inclusion of $[HCO_3^-]$ on either surface, which is inconsistent with experimental evidence. Thus, it is concluded that the proton source is not derived from water.

Therefore, the above results demonstrate that HCO_3^- is the sole proton source.

2. Here, we examine the effect of intermediate coverages on the Tafel slope and other reactant dependencies to further confirm our proposed mechanism. We also

present a thorough analysis of other potential rate-determining steps to demonstrate that the expected Tafel slopes and reaction orders with respect to HCO_3^- would not be consistent with our experimental observations, further confirming our proposed mechanism.

Given our proposed mechanism as re-written below, we can derive the expected Tafel slopes as a function of each reaction intermediate for each of the possible rate-determining steps:

$$CO_2 + e^- + * \rightarrow CO_2^{\cdot -*} \quad (8)$$

$$CO_2^{\cdot -*} + HCO_3^- + e^- \rightarrow HCOO^{-*} + CO_3^{2-} \quad (9)$$

$$HCOO^{-*} \rightarrow HCOO^- + * \quad (10)$$

With the exception of the rate-determining step, we assume all other steps are in quasi-equilibrium as given by the following expressions:

$$K_{(8)} = \frac{\theta_{CO_2^{\cdot -*}}}{P_{CO_2}\,\theta^*} \qquad K_{(9)} = \frac{\theta_{HCOO^{-*}}}{\theta_{CO_2^{\cdot -*}}\,[HCO_3^-]} \qquad K_{(10)} = \frac{[HCOO^-]\theta^*}{\theta_{HCOO^{-*}}}$$

Each K_i is an electrochemical equilibrium constant as defined by:

$$K_i = \exp(\frac{-\Delta G_i^0 - n_i F(E - E_i^0)}{RT})$$

In step (8) and (9), there is one electron transfer, and so $n_{(8),(9)}$= 1. For step (10), no electron transfer, $n_{(10)}$= 0. For each possible case of the rate-determining step, the forward electrochemical rate constant is defined as:

$$k_i = k_i^0 \exp(\frac{-\Delta G_i^0 - n_i \beta_i F(E - E_i^0)}{RT})$$

In all cases, we define the Tafel slope for a given rate expression as:

$$\text{slope} = -\frac{2.3}{\frac{\partial ln i}{\partial E}}$$

2.1 In the main text, we assert that step (9) is the rate-determining step on Co_3O_4 single-unit-cell layer, so we first show the expected Tafel slope for the overall rate expression:

$$i_{HCOO^-} = k_{(9)}\theta_{CO_2^{\cdot -*}}[HCO_3^-]$$

With the remaining steps in quasi-equilibrium, we can then write:

$$\theta_{HCOO^{-*}} = \frac{[HCOO^{-}]\theta^{*}}{K_{(10)}}$$

$$\theta_{CO_2^{\cdot-*}} = K_{(8)}P_{CO_2}\theta^{*}$$

Then, the overall rate concentration of open surface sites may be written as:

$$\theta^{*} = \frac{1}{1 + K_{(8)}P_{CO_2} + \frac{[HCOO^{-}]}{K_{(10)}}}$$

By substitution of these expressions into the rate equation and taking the derivative with respect to the potential, *E*, the slope is found to be:

$$\text{Tafel slope} = \frac{2.3RT}{F(1 + \beta_{(9)} - \theta_{CO_2^{\cdot-*}})}$$

For illustrative purposes, the complete derivation of this expression is as follows:

$$\ln(i_{HCOO^{-}}) = \ln(k_{(9)}\theta_{CO_2^{\cdot-*}}[HCO_3^{-}])$$

$$\ln(i_{HCOO^{-}}) = \ln k_{(9)} + \ln \theta_{CO_2^{\cdot-*}} + \ln[HCO_3^{-}]$$

$$= \ln k_{(9)} + \ln[HCO_3^{-}] + lnK_{(8)} + lnP_{CO_2} + ln\theta^{*}$$

$$\frac{\partial \ln(i_{HCOO^{-}})}{\partial E} = \frac{\partial}{\partial E}\left(\ln k_{(9)} + \ln[HCO_3^{-}] + lnK_{(8)} + lnP_{CO_2} + ln\theta^{*}\right)$$

$$= \frac{\partial}{\partial E}\left(\ln k_{(9)} + lnK_{(8)} + ln\theta^{*}\right)$$

$$= -\frac{\beta_{(9)}F}{RT} - \frac{F}{RT} + \frac{\partial}{\partial E}\ ln\theta^{*}$$

$$= -\frac{\left(1 + \beta_{(9)}\right)F}{RT} - \frac{\partial}{\partial E} ln\frac{1}{\theta^{*}}$$

$$= -\frac{\left(1 + \beta_{(9)}\right)F}{RT} - \theta^{*}\frac{\partial(\frac{1}{\theta^{*}})}{\partial E}$$

$$\frac{\partial \ln(i_{HCOO^{-}})}{\partial E} = -\frac{\left(1 + \beta_{(9)}\right)F}{RT} - \theta^{*}\frac{\partial}{\partial E}\left(1 + K_{(8)}P_{CO_2} + \frac{[HCOO^{-}]}{K_{(10)}}\right)$$

$$= -\frac{\left(1 + \beta_{(9)}\right)F}{RT} - \theta^{*}\left(\frac{F}{RT}\right)\left(-K_{(8)}P_{CO_2}\right)$$

$$= -\frac{F}{RT}(1 + \beta_{(9)} - \theta_{CO_2^{\cdot-*}})$$

$$\text{Tafel slope} = -\frac{2.3}{\frac{\partial \ln(i_{HCOO^{-}})}{\partial E}} = \frac{2.3RT}{F(1 + \beta_{(9)} - \theta_{CO_2^{\cdot-*}})}$$

Here, we assume that the $\beta_{(9)}$ = 0.5 and $\theta_{CO_2^{\cdot -*}} \ll 1$. As derived, the Tafel slope should then be 39 mV dec^{-1} at $\theta_{CO_2^{\cdot -*}} \ll 1$, which we assume to be true in the kinetically-limited, low-current regime on the V_o-rich Co_3O_4 single-unit-cell layer. This low-current regime is further supported by order and linearity of the dependence studies, as shown in the main text. Also, the form of this Tafel slope expression also supports our observations on V_o-poor Co_3O_4 single-unit-cell layer, with an expected Tafel slope of 48 mV dec^{-1} at $\theta_{CO_2^{\cdot -*}} = 0.27$.

Therefore, the above results demonstrate that the V_o-rich Co_3O_4 single-unit-cell layer has a surface coverage of $CO_2^{\cdot -*} \ll 1$, while the V_o-poor Co_3O_4 single-unit-cell layer has $\theta_{CO_2^{\cdot -*}} = 0.27$.

2.2 If we assume instead that the 1st step, (8), is rate-determining, then:

$$i_{HCOO^-} = k_{(8)} P_{CO_2} [HCO_3^-]$$

$$\theta_{HCOO^{-*}} = \frac{[HCOO^-]\theta^*}{K_{(10)}}$$

$$\theta_{CO_2^{\cdot -*}} = \frac{[CO_3^{2-}]\theta_{HCOO^{-*}}}{K_{(9)}[HCO_3^-]} = \frac{[CO_3^{2-}]\,[HCOO^-]\theta^*}{K_{(9)}K_{(10)}[HCO_3^-]}$$

$$\theta^* = \frac{1}{1 + \frac{[HCOO^-]\,[CO_3^{2-}]}{K_{(9)}K_{(10)}[HCO_3^-]} + \frac{[HCOO^-]}{K_{(10)}}}$$

$$\text{Tafel slope} = \frac{2.3RT}{F(\beta_{(8)} + \theta_{CO_2^{\cdot -*}})}$$

In this case, we can see that the Tafel slope at low coverage of the $CO_2^{\cdot -*}$ intermediates would be 118 mV dec^{-1}, assuming $\beta_{(8)} = 0.5$. With increasing $\theta_{CO_2^{\cdot -*}}$, the Tafel slope would be expected to decrease: first to 59 mV dec^{-1} at $\theta_{CO_2^{\cdot -*}} = 0.5$, then down to 39 mV dec^{-1} at $\theta_{CO_2^{\cdot -*}} = 1$. We find this behavior to be counterintuitive, and the necessity of moderate to high coverage to match our observed Tafel slope on Co_3O_4 single-unit-cell layer does not agree with our reaction order studies for

$[HCO_3^-]$.

2.3 Finally, if the 3th step, (10), were rate-determining, then the following is the derivation of the Tafel slope:

$$i_{HCOO^-} = k_{(10)}\theta_{HCOO^{-*}}$$

$$\theta_{CO_2^{\cdot -*}} = K_{(8)}P_{CO_2}\theta^*$$

$$\theta_{HCOO^{-*}} = \frac{K_{(9)}[HCO_3^-]\theta_{CO_2^{\cdot -*}}}{[CO_3^{2-}]} = \frac{K_{(8)}K_{(9)}[HCO_3^-]P_{CO_2}\theta^*}{[CO_3^{2-}]}$$

$$\theta^* = \frac{1}{1 + K_{(8)}P_{CO_2} + \frac{K_{(8)}K_{(9)}[HCO_3^-]P_{CO_2}}{[CO_3^{2-}]}}$$

$$\text{Tafel slope} = \frac{2.3RT}{F(2 - \theta_{CO_2^{\cdot -*}} - 2\theta_{HCOO^{-*}})}$$

Notably, this is the only choice of the rate-determining step which results in an explicit dependence on $\theta_{HCOO^{-*}}$. At low coverage, however, the expected value is 30 mV dec^{-1}, which is lower than the value we observe at low overpotentials on Co_3O_4 single-unit-cell layer. For this to be the correct rate-determining step, then we would need to be operating at intermediate coverage of $\theta_{HCOO^{-*}}$ to obtain our observed Tafel slope of 37 and 48 mV dec^{-1}. In that region, however, the necessity of moderate to low coverage to match our observed Tafel slope on Co_3O_4 single-unit-cell layer does not agree with our reaction order studies for $[HCO_3^-]$.

Briefly, the above results could be concluded as follows:

(1) The reaction pathway was

$CO_2 + e^- + * \rightarrow CO_2^{\cdot -*}$ (8)

$CO_2^{\cdot -*} + HCO_3^- + e^- \rightarrow HCOO^{-*} + CO_3^{2-}$ (9)

$HCOO^{-*} \rightarrow HCOO^- + *$ (10)

(2) HCO_3^- is the sole proton source, while the H^+ donation from HCO_3^- was the rate-limiting step;

(3) The V_o-rich Co_3O_4 single-unit-cell layer has a surface coverage of $CO_2^{\cdot -*} \ll 1$, while the V_o-poor Co_3O_4 single-unit-cell layer has $\theta_{CO_2^{\cdot -*}} = 0.27$.

To evaluate the effective U parameters, we further calculated the formation energy of oxygen vacancy (Supplementary Table 5) and the energetic scheme with differently effective U values adopted in previous works including: 2.0[23-27], 3.3[28], *3.5*[29,30] with 2.5 for comparison. The number of electrons needed to adjust the work function is fitted as the method described above and the free energy potential diagrams are also calculated as shown in the following Supplementary Table 5 and Supplementary Figure 13. It is suggested that the decreasing value of U_{eff} greatly reduced the free energy change of the hydrogen attachment step but not changed the trend that the free energy change on the V_o-rich Co_3O_4 single-unit-cell layers is lower than that on the V_o-poor Co_3O_4 single-unit-cell layers.

Of note, the relative free energy especially for U_{eff}=2.0 in the second step becomes exothermic. Thus, it is suggested that U_{eff} should not be too small. For U_{eff} = 2.5, 3.3, and 3.5, all the results show that the second step is the rate limiting step, and more importantly, the free energy change on the V_o-rich Co_3O_4 single-unit-cell layers is lower than that on the V_o-poor Co_3O_4 single-unit-cell layers, in which the difference is >0.10 eV with all the U_{eff} values even with U_{eff}=2.0. Hence, the results with U_{eff}=3.5 are given in the main text.

The most important reason for which we finally choose U_{eff}=3.5 is shown as follows: according to the above kinetic analysis, the coverage of CO_2^{-}* on V_o-poor Co_3O_4 single-unit-cell layers (0.27) under real condition is larger than that on V_o-rich Co_3O_4 single-unit-cell layer(<<1), which indicates the former's adsorption energy of CO_2^{-}* under real condition is larger than that on the latter based on quasi-equibrium assumption. Among all the U_{eff} values we tested, only U_{eff} = 3.5 gives the right order of the adsorption energy of CO_2^{-}* at real condition as listed in the following Supplementary Table 5. Thus finally, U_{eff} = 3.5 is adopted. With U_{eff}=3.5, the electrons should be added to 0.92 e^- and 0.62 e^- for V_o-poor and V_o-rich Co_3O_4 single-unit-cell layers. The finally obtained free energy difference on two sample surfaces at real overpotential is 0.11 eV.

Supplementary References

1. Käckell, P., Furthmüller, J., Bechstedt, F., Kresse, G. & Hafner, J. Characterization of carbon-carbon bonds on the SiC(001)c(2×2) surface. *Phys. Rev. B.* **54**, 304-307 (1996).
2. Kresse, G. & Furthmüller, J. Efficiency of ab-initio total energy calculations for metals and semiconductors using a plane-wave basis set. *Comp. Mater. Sci.* **6**, 15-50 (1996).
3. García-Mota, M. et al. Importance of correlation in determining electrocatalytic oxygen evolution activity on cobalt oxides. *J. Phys. Chem. C.* **116**, 21077-21082 (2012).
4. Mortensen, J. J., Hansen, L. B. & Jacobsen, K. W. Real-space grid implementation of the projector augmented wave method. *Phys. Rev. B***.** **71**, 1-11 (2005).
5. Perdew, John P., Burke, K. & Ernzerhof, M. Generalized gradient approximation made simple. *Phys. Rev. Lett.* **77**, 3865-3868 (1996).
6. Methfessel, M. & Paxton, A. High-precision sampling for Brillouin-zone integration in metals. *Phys. Rev. B.* **40**, 3616-3621 (1989).
7. Monkhorst, H. J. & Pack, J. D. Special points for Brillonin-zone integrations. *Phys. Rev. B.* **13**, 5188-5192 (1976).
8. Schaub, R. et al. Oxygen Vacancies as Active Sites for Water Dissociation on Rutile TiO_2(110). *Phys. Rev. Lett.* **87**, (2001).
9. Nørskov, J. K. et al.Origin of the overpotential for oxygen reduction at a fuel-cell cathode. *J. Phys. Chem. B.* **108**, 17886-17892 (2004**).**
10. Tsiplakidesa, D., Archontab, D., & Vayenas, C. G. Absolute potential measurements in solid and aqueous electrochemistry using two Kelvin probes and their implications for the electrochemical promotion of catalysis. *Top. Catal.* **44**, 469-479(2007).
11. Fang, Y. H. & Liu, Z. P. Surface Phase Diagram and Oxygen Coupling Kinetics on Flat and Stepped Pt Surfaces under Electrochemical Potentials. *J. Phys. Chem. C.* **113**, 9765-9772 (2009).
12. Fang, Y. H. & Liu, Z. P. Mechanism and Tafel Lines of Electro-Oxidation of Water to Oxygen on RuO_2(110). *J. Am. Chem. Soc.* **132**, 18214-18222 (2010)
13. Vesselli, E. et al. Steering the Chemistry of Carbon Oxides on a NiCu Catalyst. *ACS Catal*. **3**, 1555-1559 (2013).
14. Bronsted, N. Acid and Basic catalysis. *Chem. Rev.* **5**, 231-338 (1928**).**
15. Rosen, J. et al. Mechanistic Insights into the Electrochemical Reduction of CO_2 to CO on Nanostructured Ag Surfaces. *ACS Catal*. **5**, 4293-4299 (2015).
16. Salehi-Khojin, A. et al. Nanoparticle Silver Catalysts That Show Enhanced Activity for Carbon Dioxide Electrolysis. *J. Phys. Chem. C.* **117**, 1627-1632 (2013).
17. Gao, S. et al. Ultrathin Co_3O_4 Layers Realizing Optimized CO_2 Electroreduction to Formate. *Angew. Chem. Int. Ed*. **55**, 698-702 (2016).
18. Li, C. W., Ciston, J. & Kanan, M. W. Electroreduction of carbon monoxide to liquid fuel on oxide-derived nanocrystalline copper. *Nature*. **508**, 504-507 (2014).
19. Gao, S. et al. Partially oxidized atomic cobalt layers for carbon dioxide electroreduction to liquid fuel. *Nature.* **529**, 68-72 (2016).

20. Chen, Y. H., Li, C. W. & Kanan, M. W. Aqueous CO_2 reduction at very low overpotential on oxide-derived Au nanoparticles. *J. Am. Chem. Soc.* **134**, 19969-19972 (2012).
21. Li, C. W. & Kanan, M. W. CO_2 reduction at low overpotential on Cu electrodes resulting from the reduction of thick Cu_2O films. *J. Am. Chem. Soc.* **134**, 7231-7234 (2012).
22. Chen, Y. H. & Kanan, M. W. Tin oxide dependence of the CO_2 reduction efficiency on tin electrodes and enhanced activity for Tin/Tin oxide thin-film catalysts. *J. Am. Chem. Soc.* **134**, 1986-1989 (2012).
23. Hu, W. D., Lan, J. G., Guo, Y., Cao, X. M. & Hu. P. Origin of Efficient Catalytic Combustion of Methane over Co_3O_4 (110): Active Low-Coordination Lattice Oxygen and Cooperation of Multiple Active Sites. *ACS Catal.* **6**, 5508-5519 (2016).
24. Fung, V., Tao, F. & Jiang, D. E. Understanding oxidative dehydrogenation of ethane on Co_3O_4 nanorods from density functional theory. *Catal. Sci. Technol*. **6**, 6861-6869 (2016).
25. Pang, X. Y. et al. Structure Sensitivity of CO Oxidation on Co_3O_4: A DFT Study. *ChemPhysChem*. **14**, 204-212 (2013).
26. Wang, H. F. et al. Origin of extraordinarily high catalytic activity of Co_3O_4 and its morphological chemistry for CO oxidation at low temperature. *J. Catal*. **296**, 110-119 (2012).
27. Lv, C. Q., Liu, C. & Wang, G. C. A DFT study of methanol oxidation on Co_3O_4. *Catal. Commun*. **45**, 83-90 (2014).
28. Cheng, F. Y. et al. Rapid room-temperature synthesis of nanocrystalline spinels as oxygen reduction and evolution electrocatalysts. *Nature Chem*. **3**, 79-84 (2011).
29. Zhang, P. X. et al. First-Principles Study of Oxygen Evolution Reaction on the Oxygen-Containing Species Covered CoII-Exposing Co_3O_4 (100) Surface. *Catal Lett*. **145**, 1169-1176 (2015).
30. García-Mota, M. et al. Importance of Correlation in Determining Electrocatalytic Oxygen Evolution Activity on Cobalt Oxides. *J. Phys. Chem. C.* **116**, 21077-21082 (2012).
